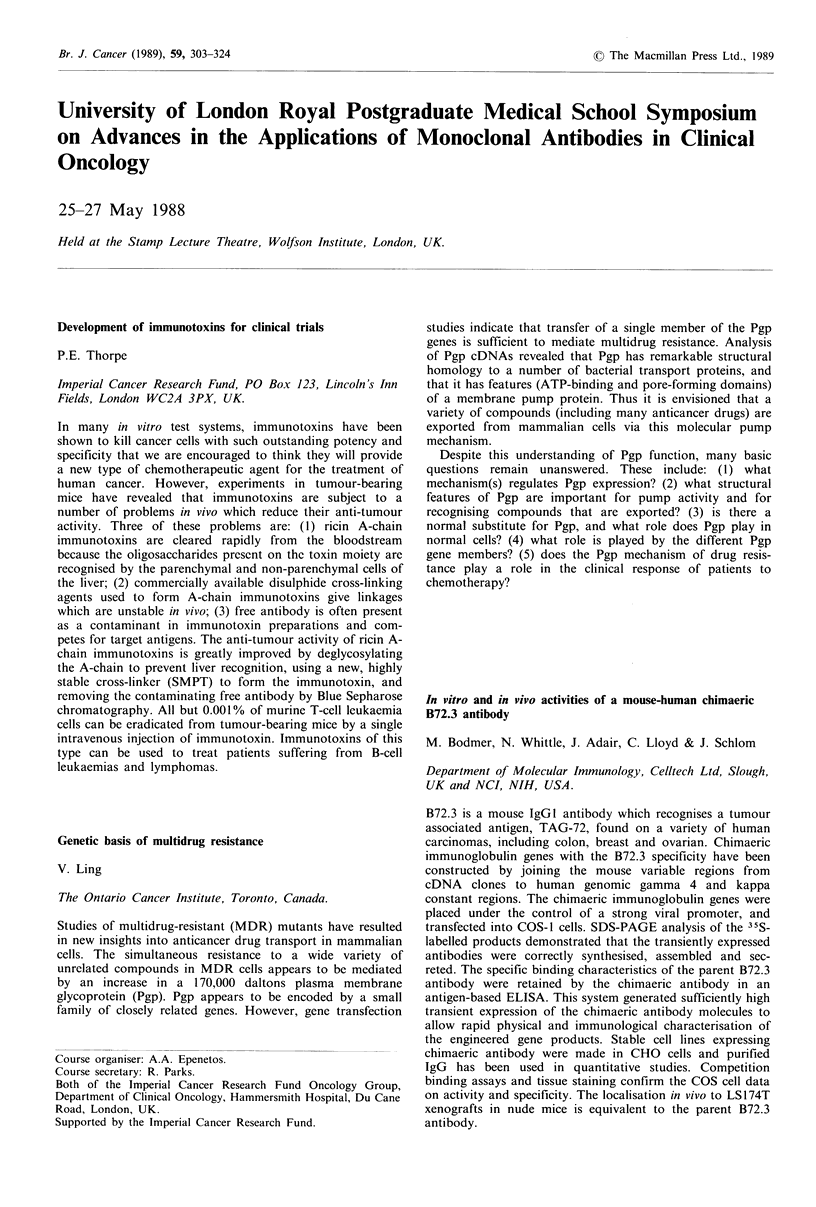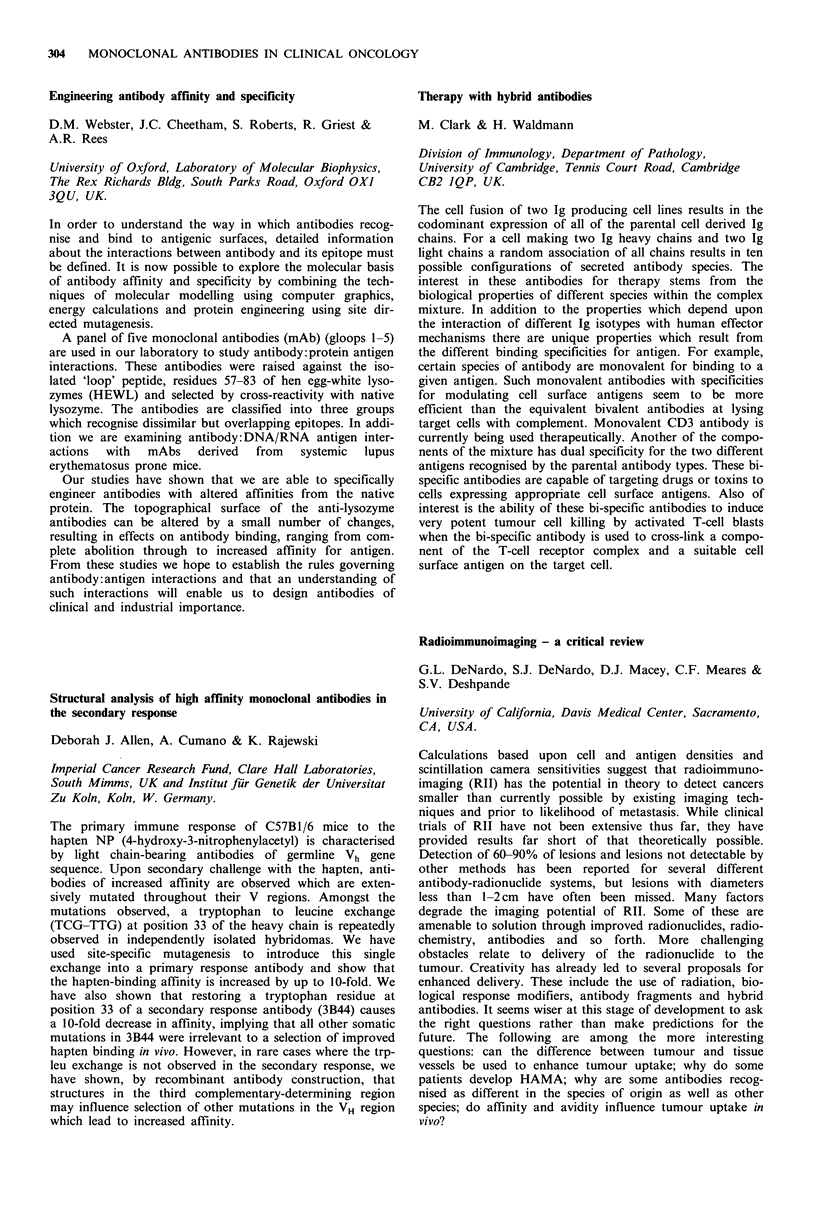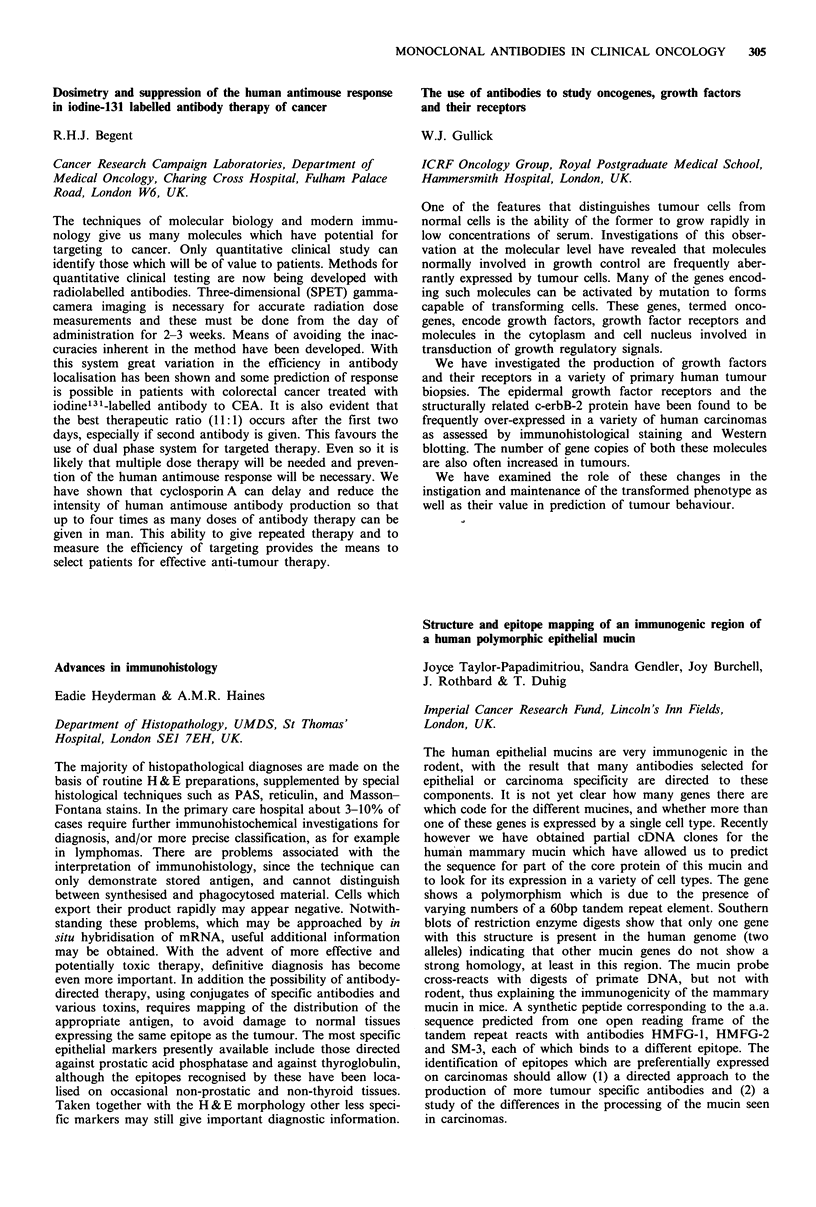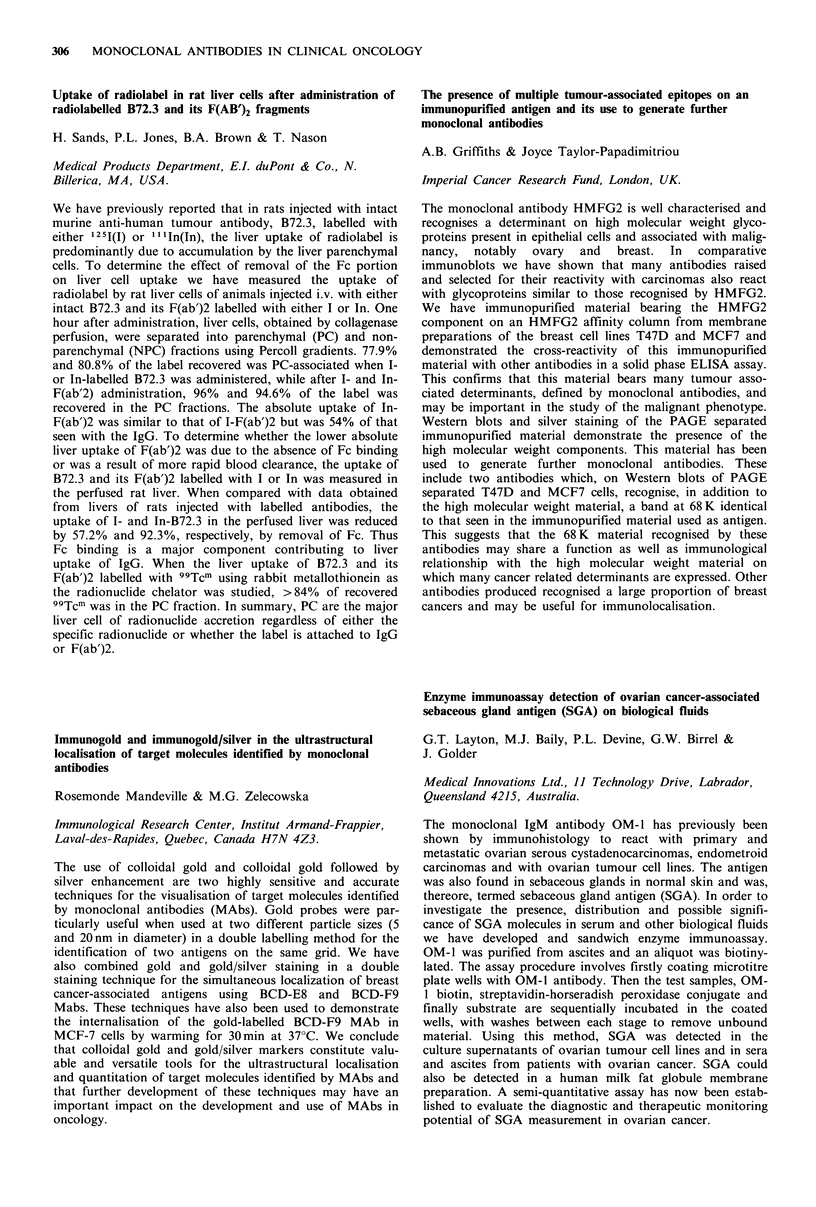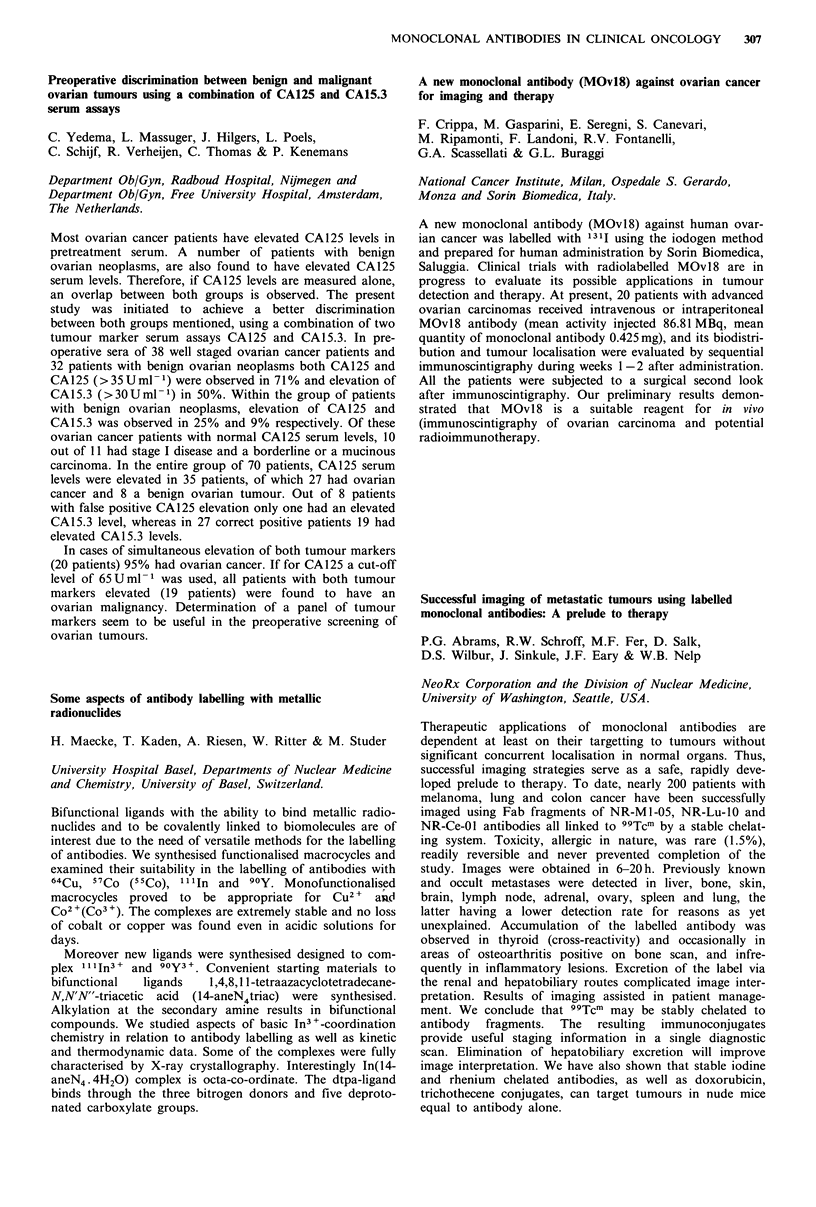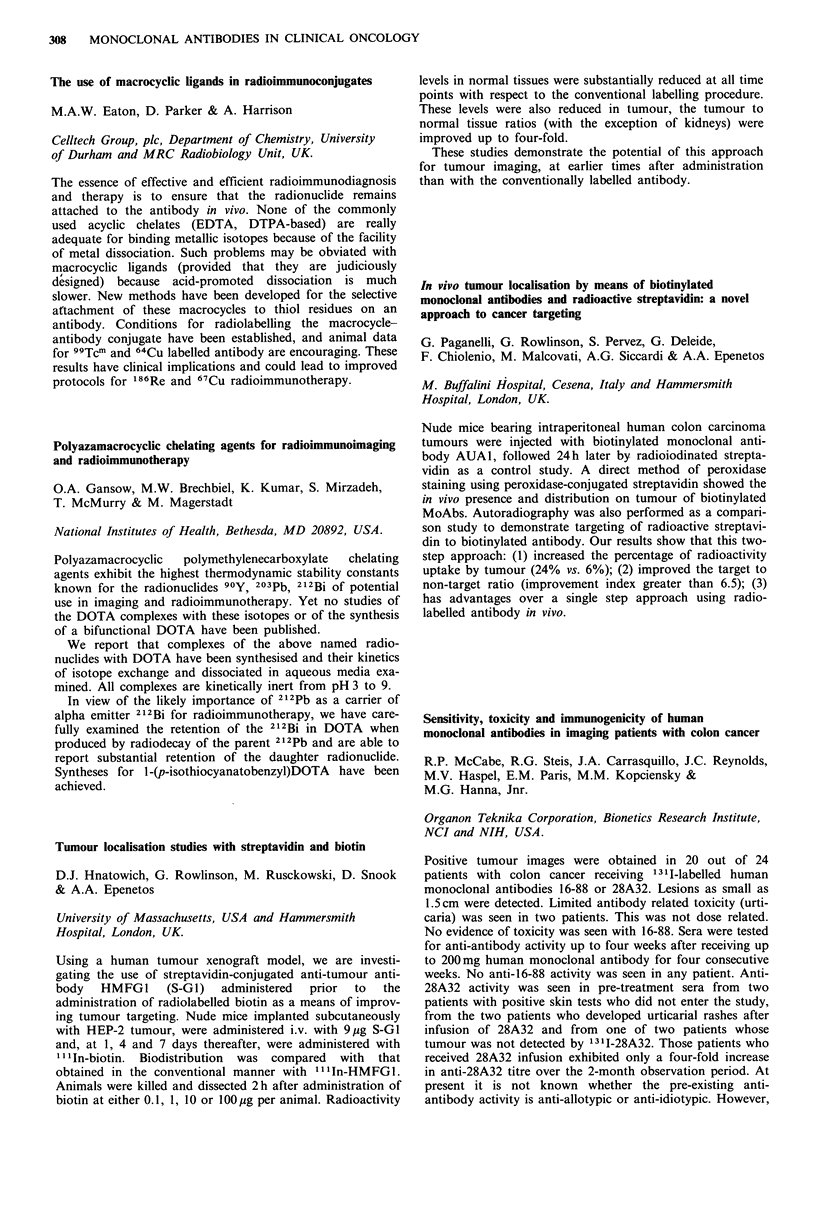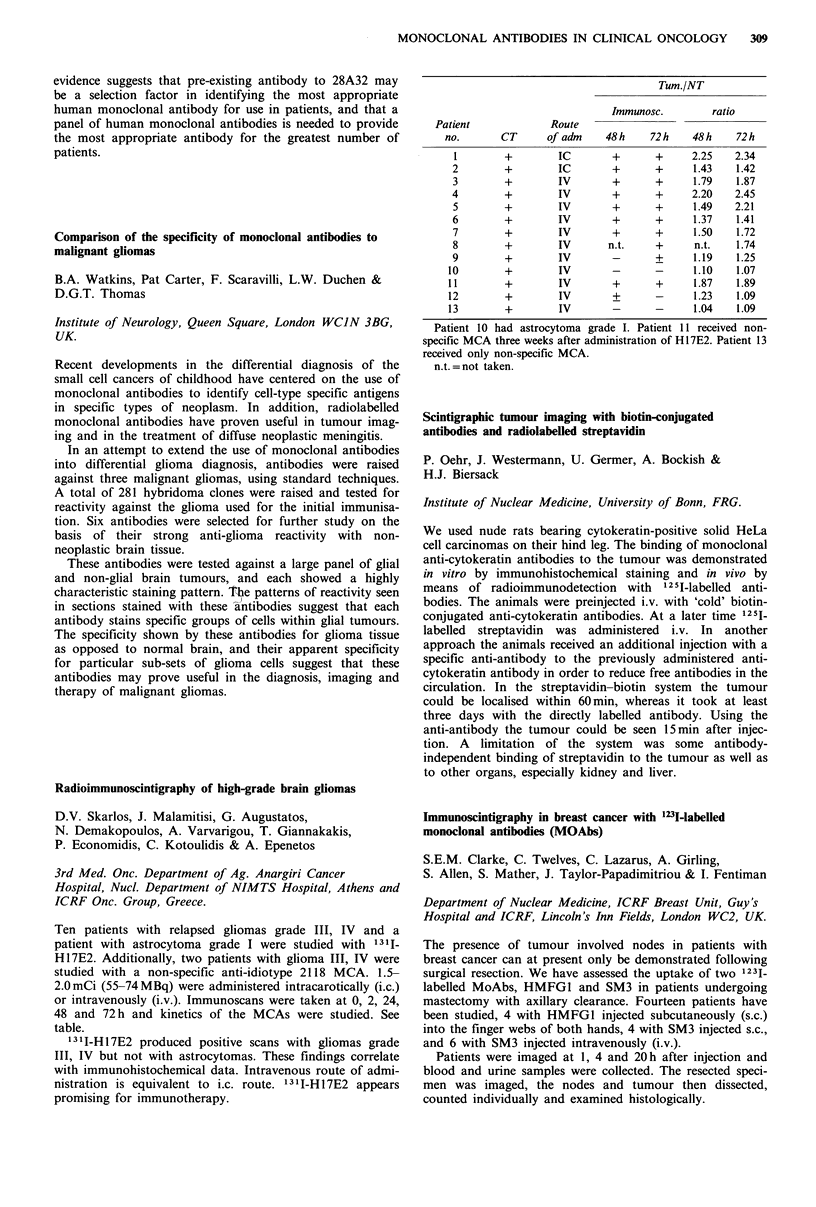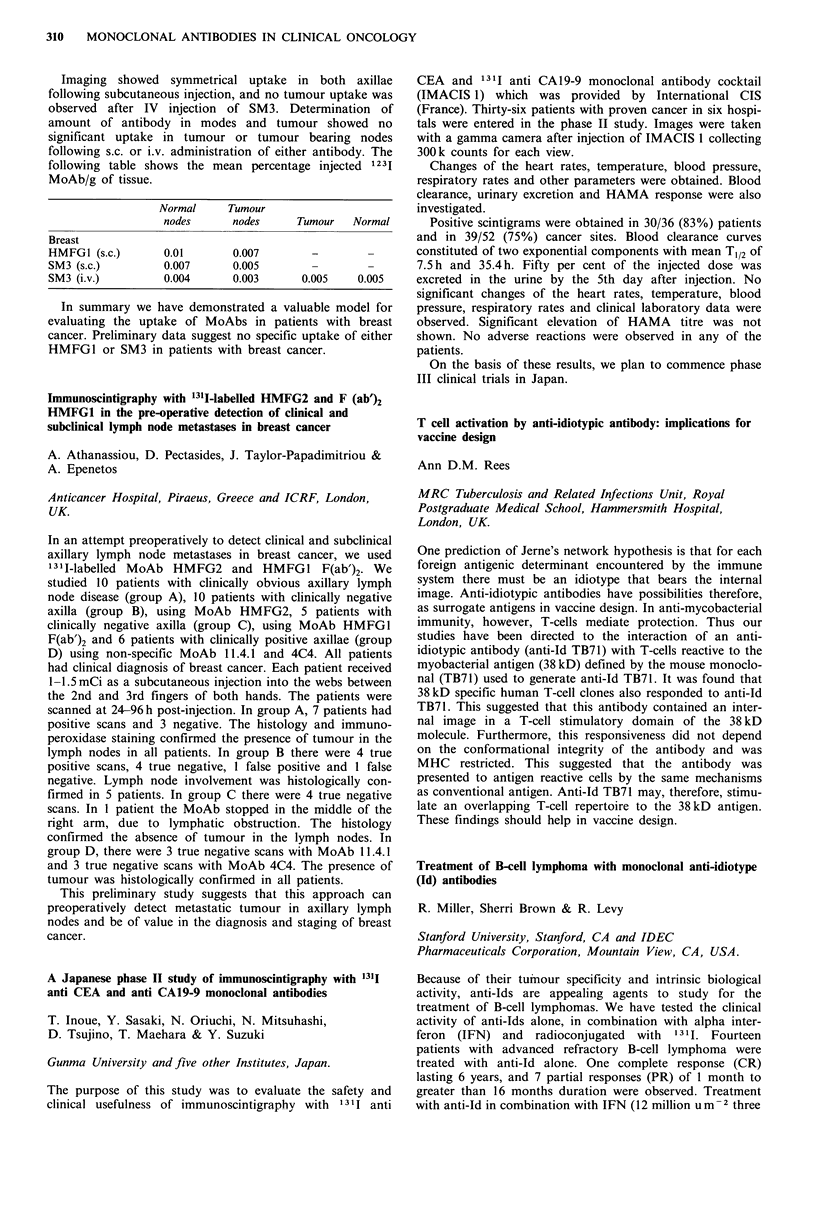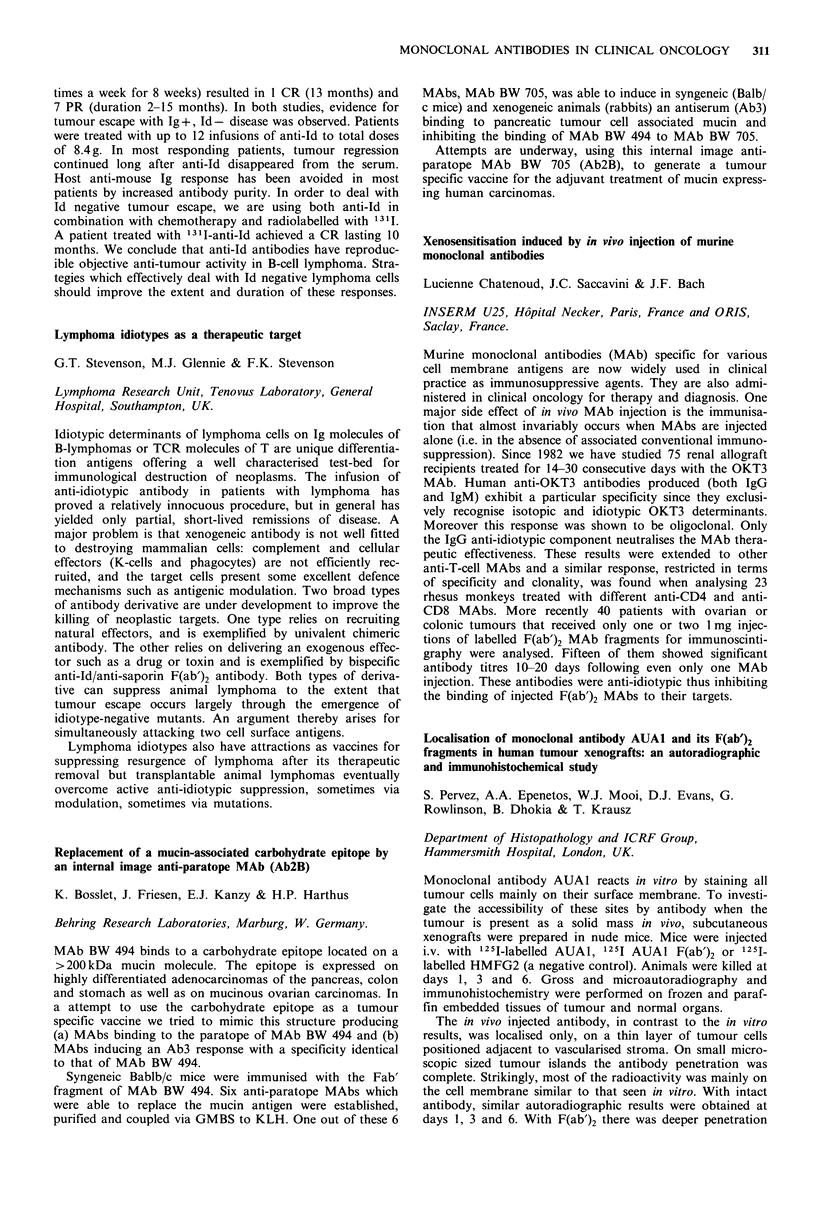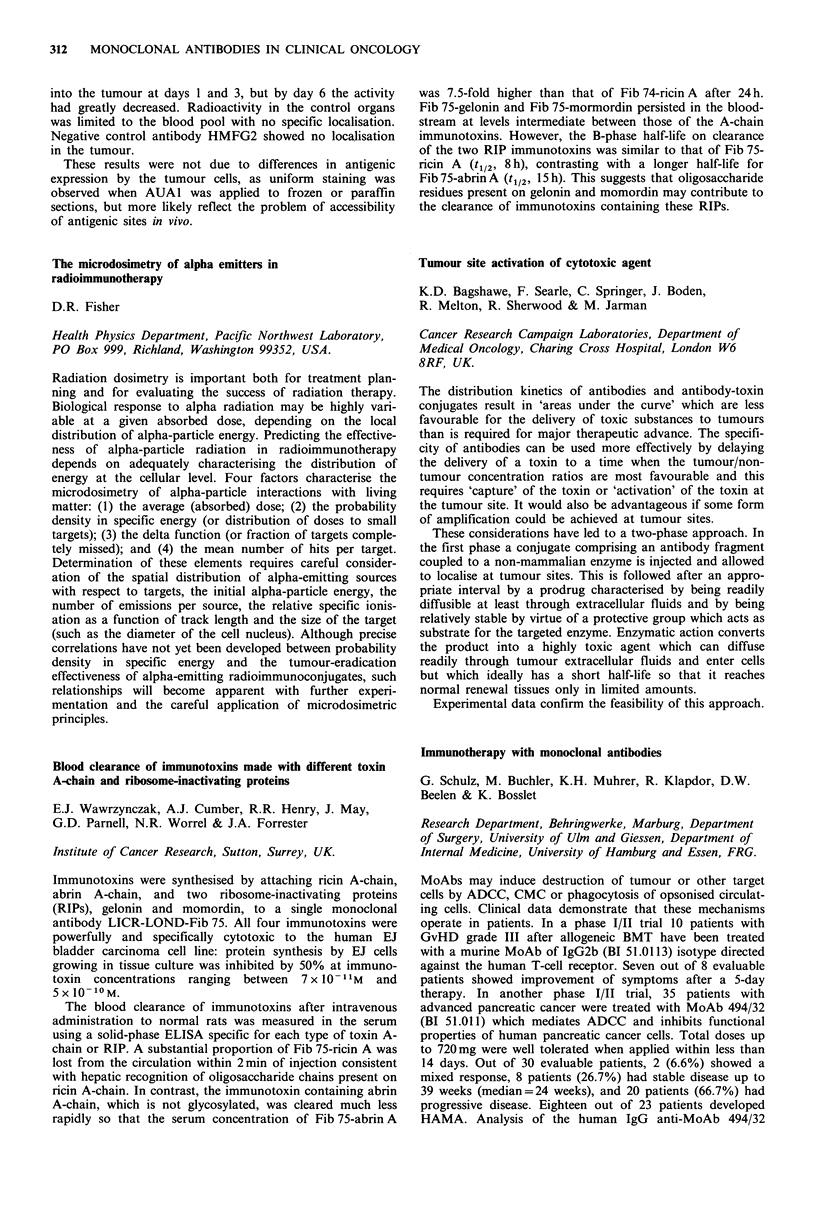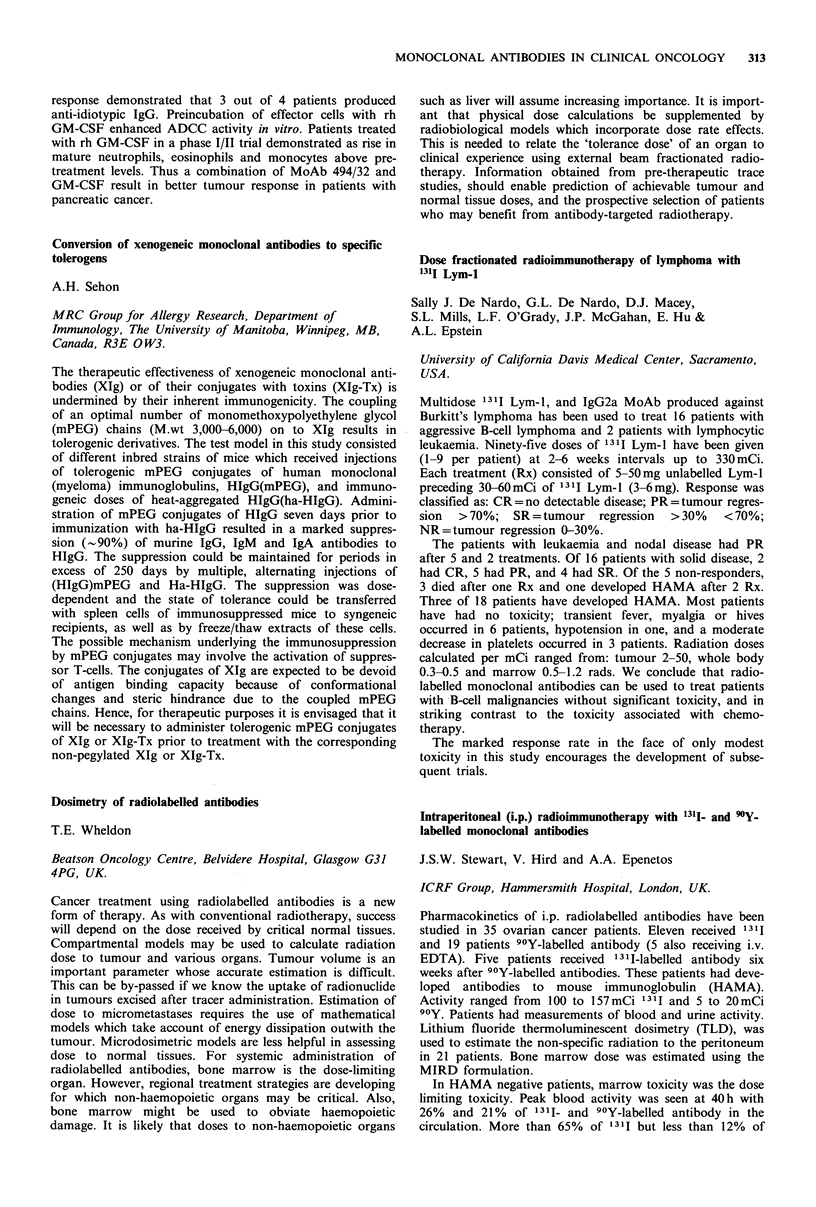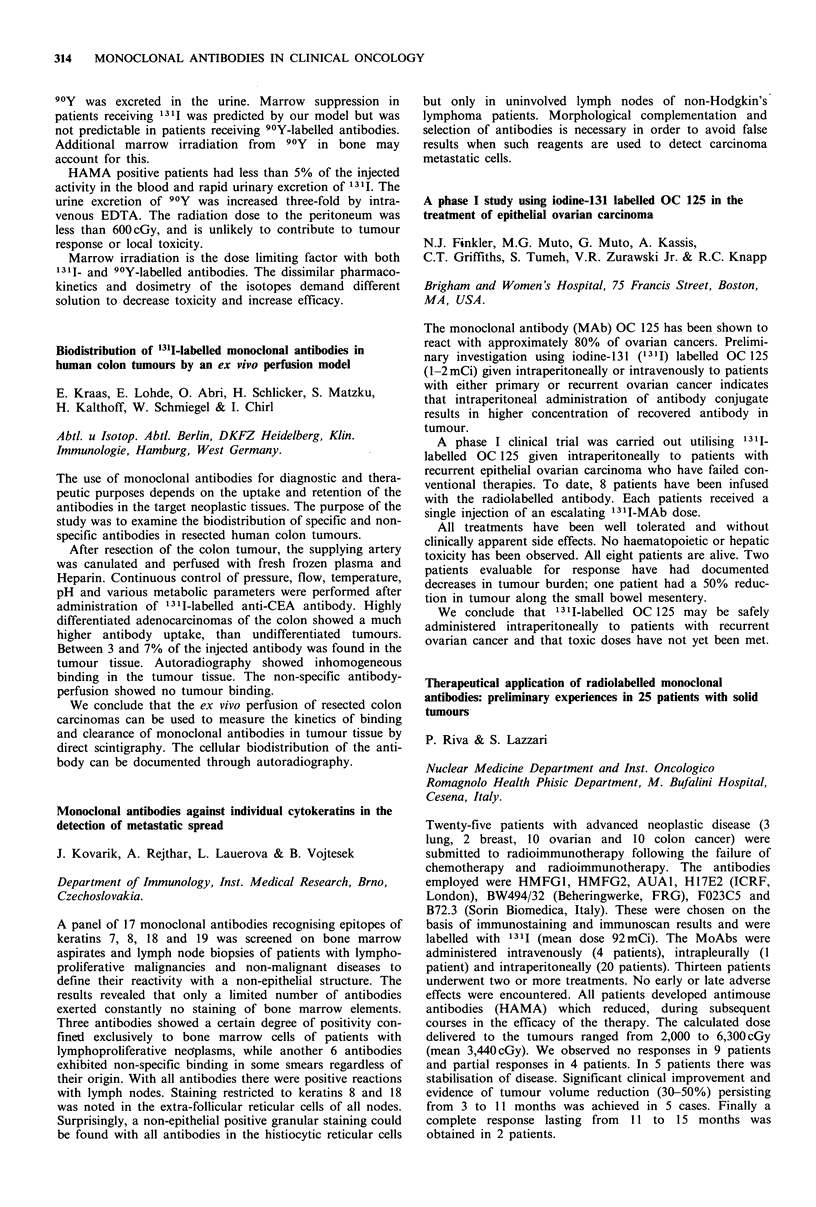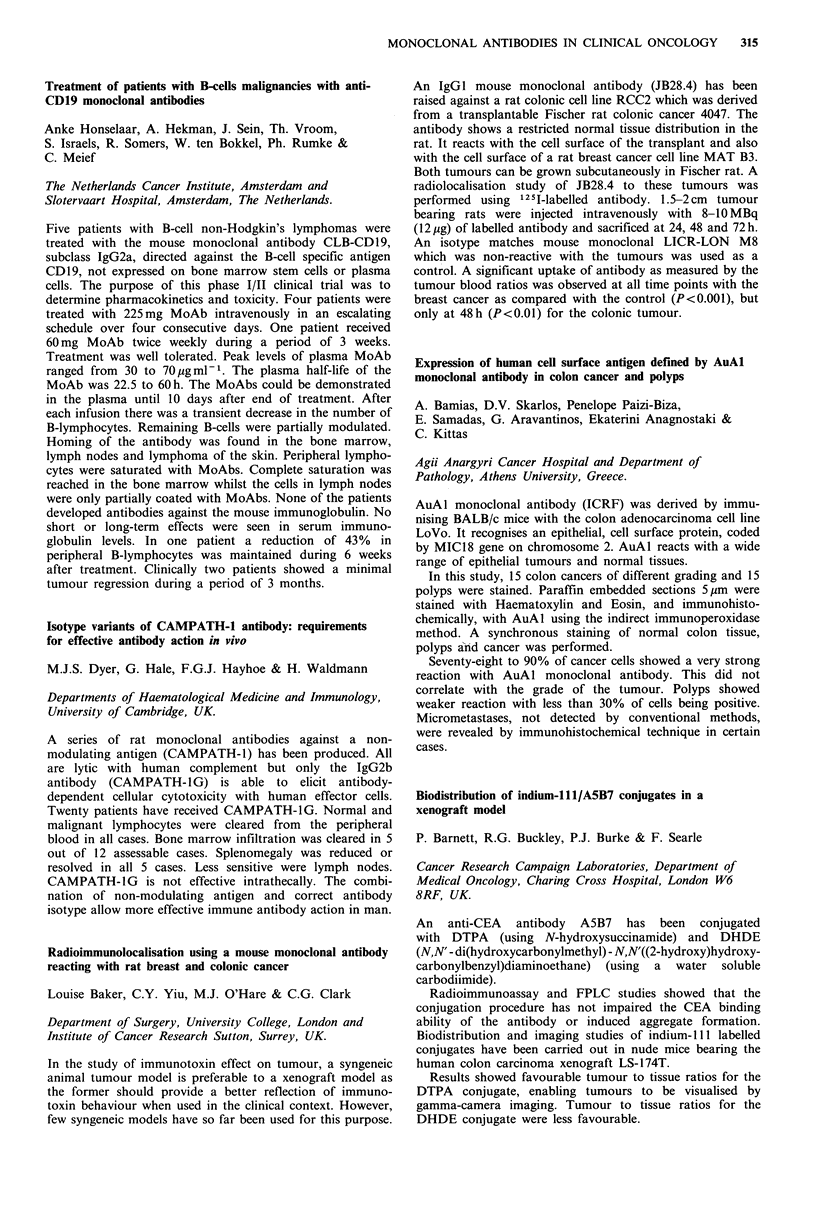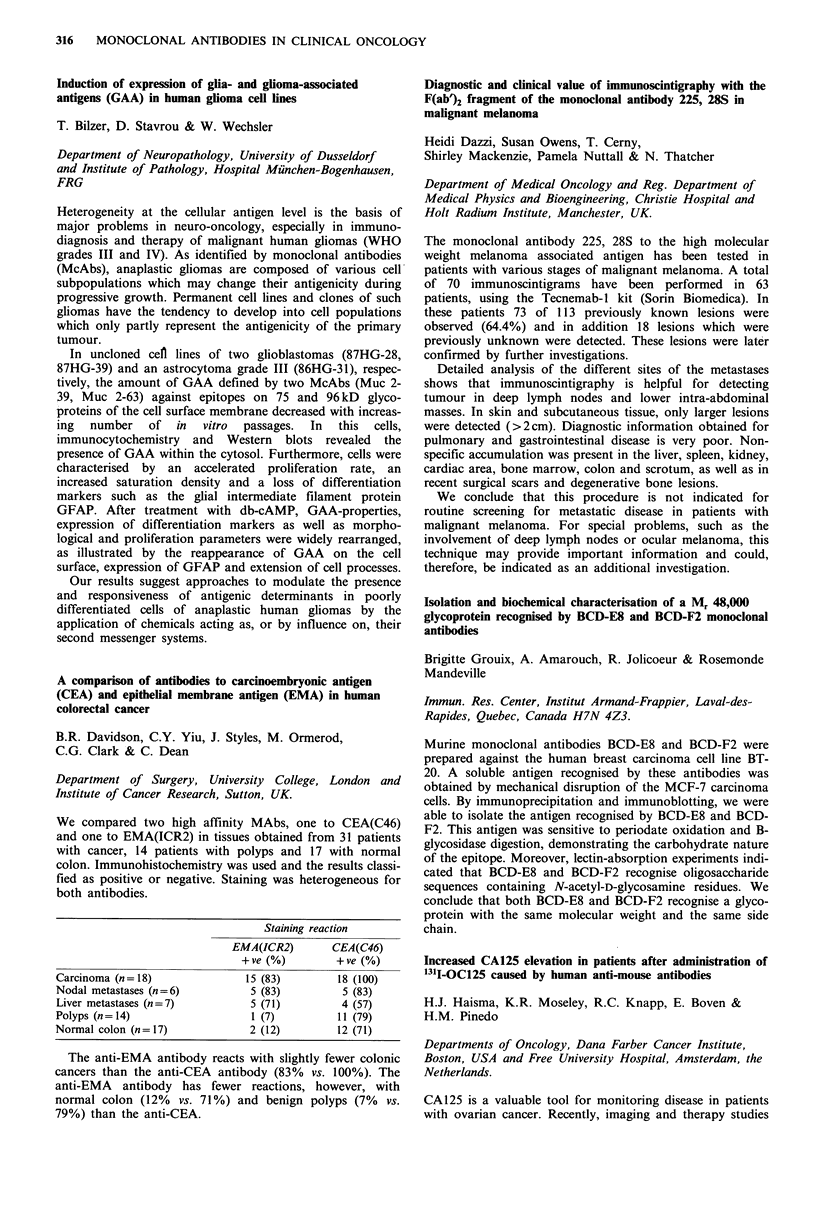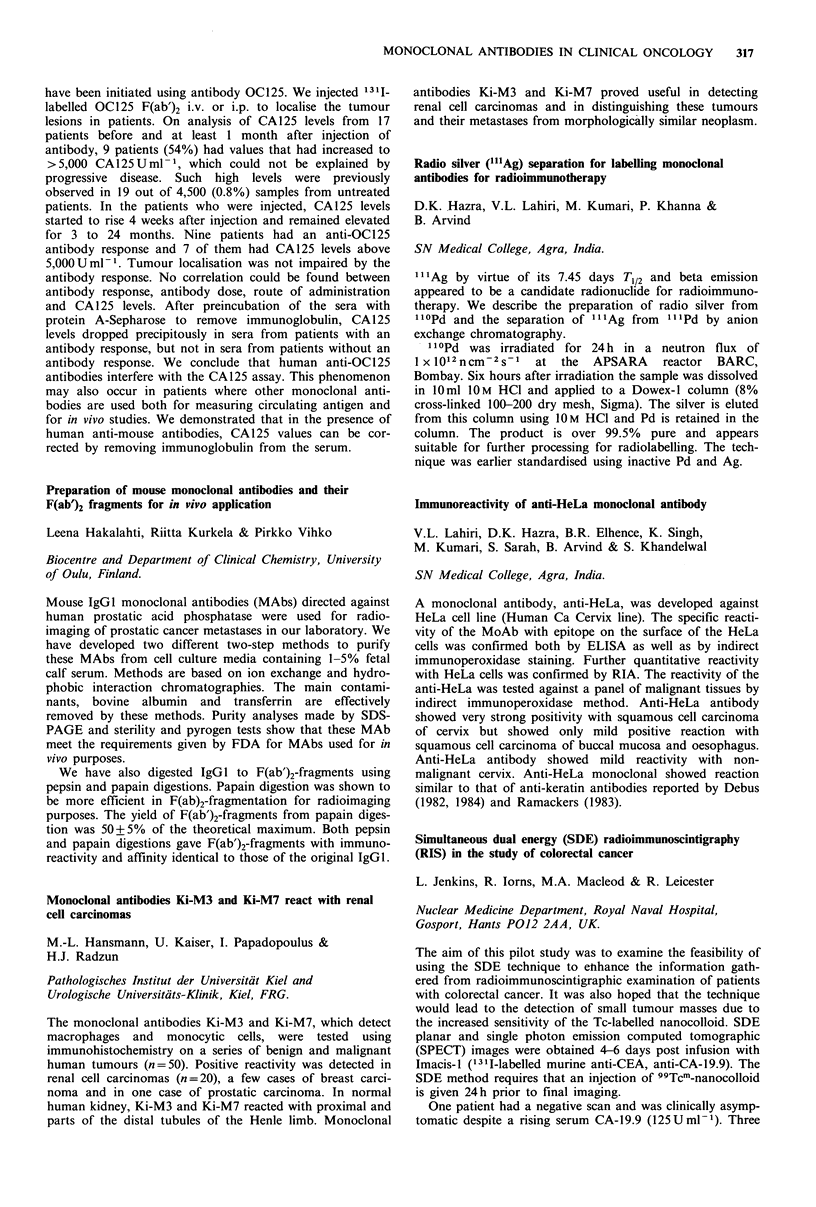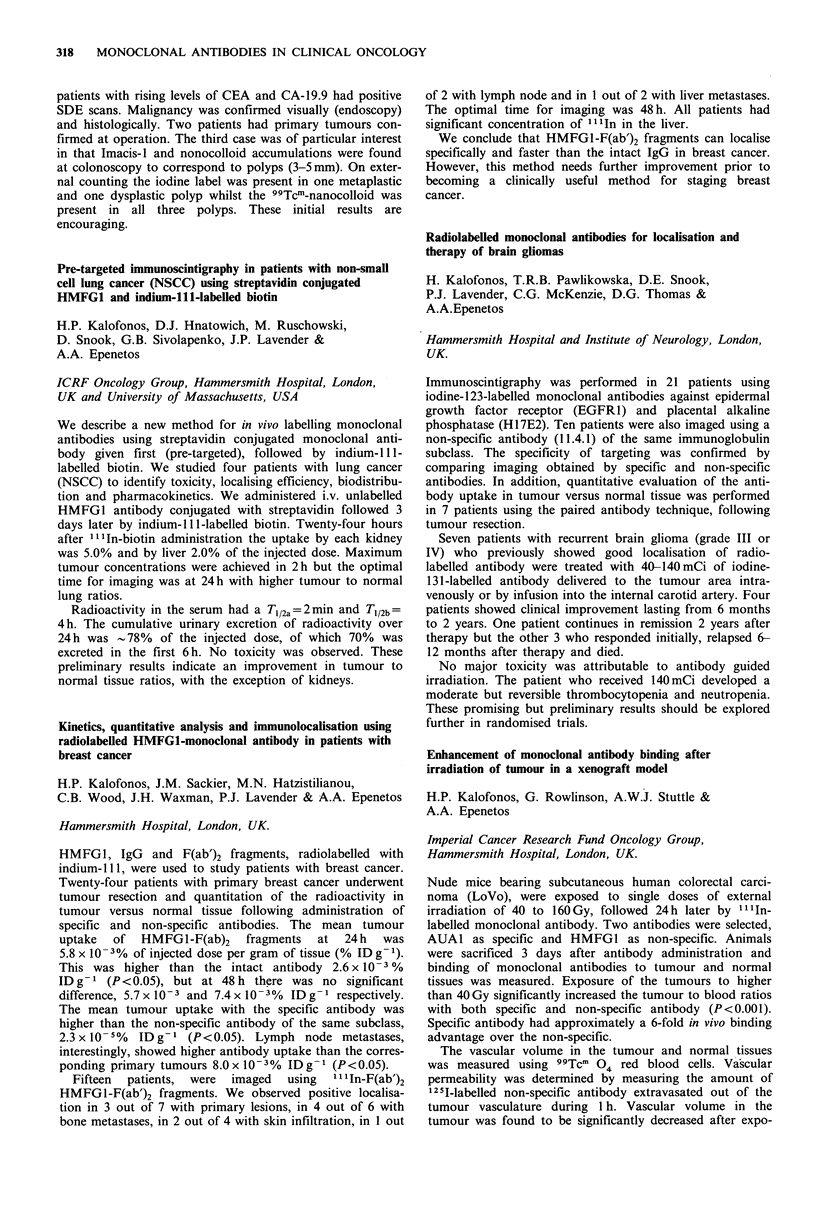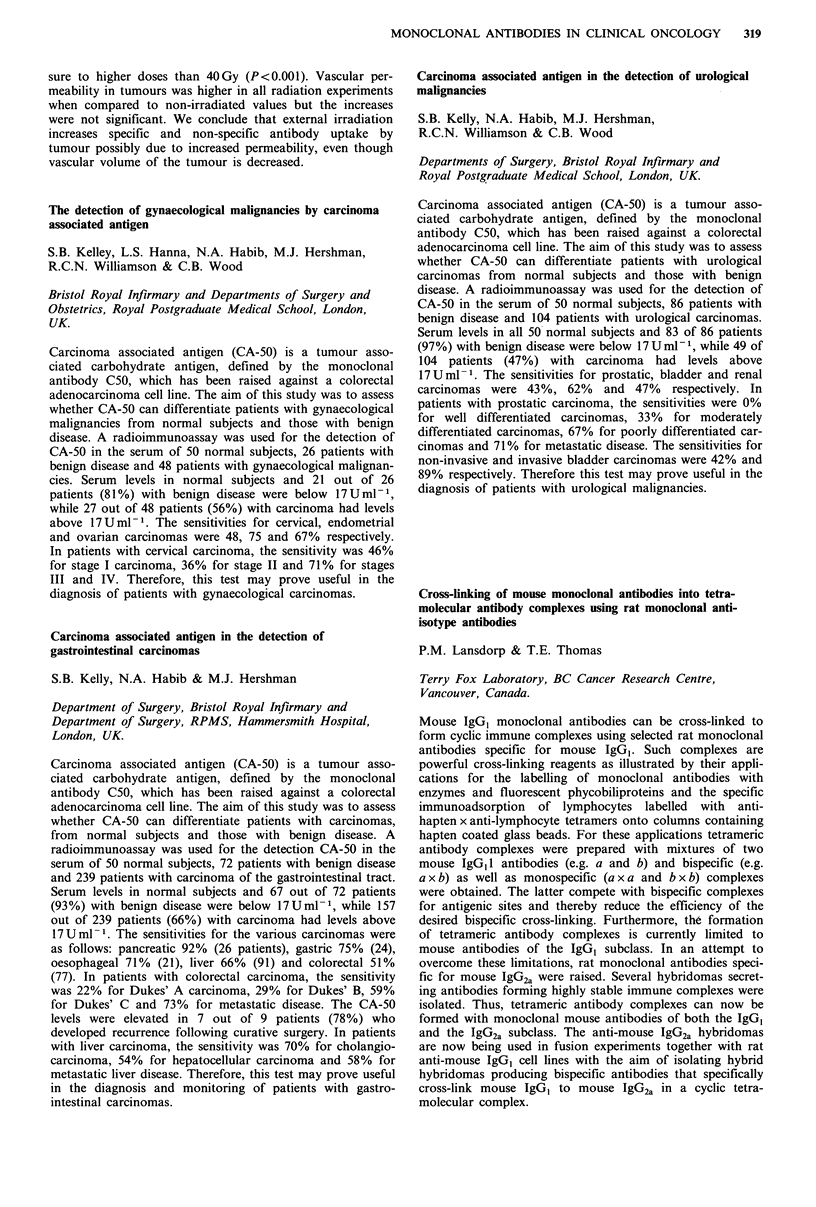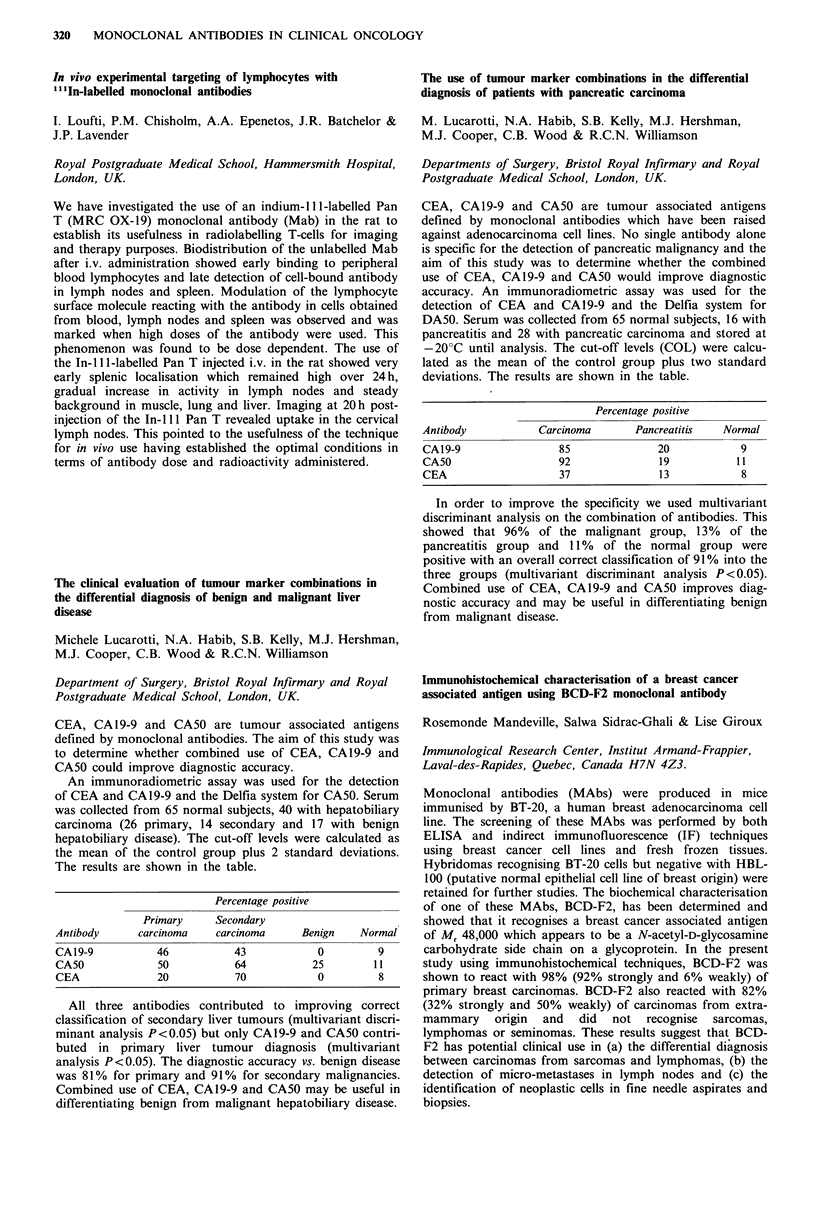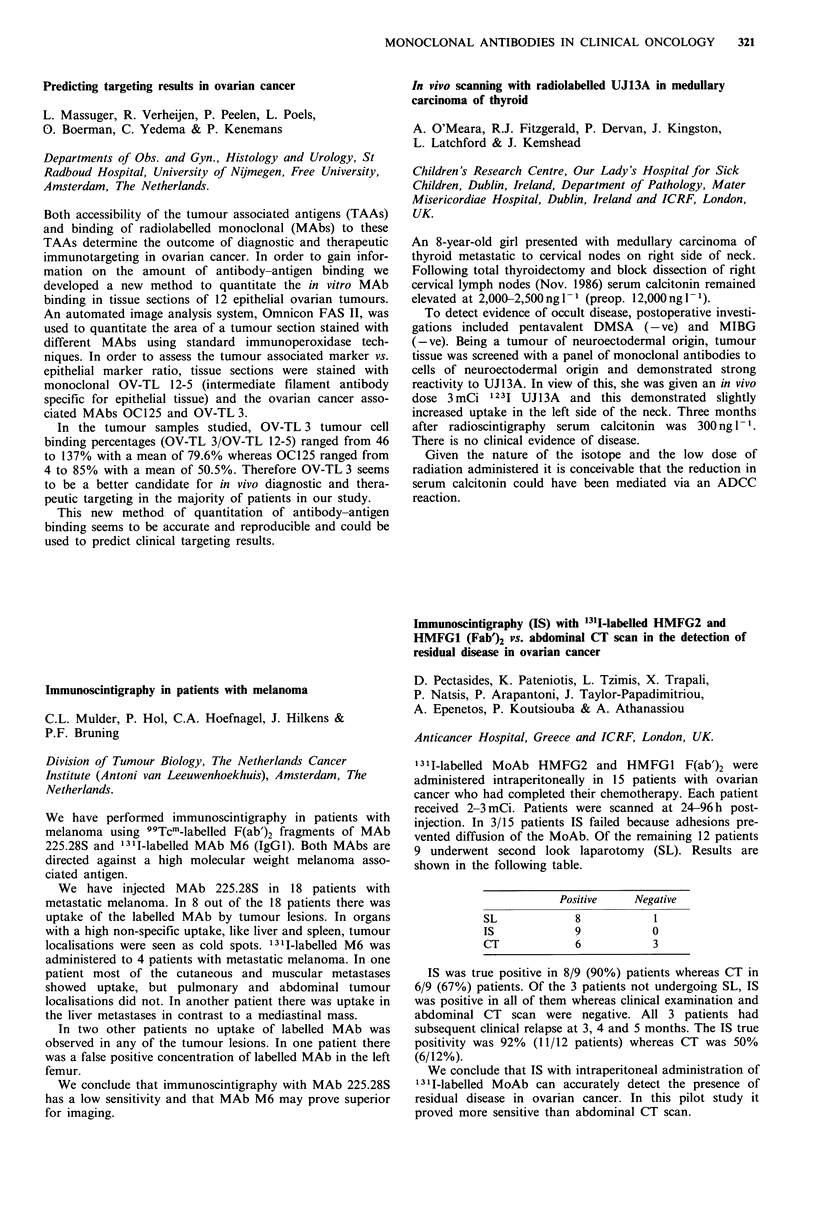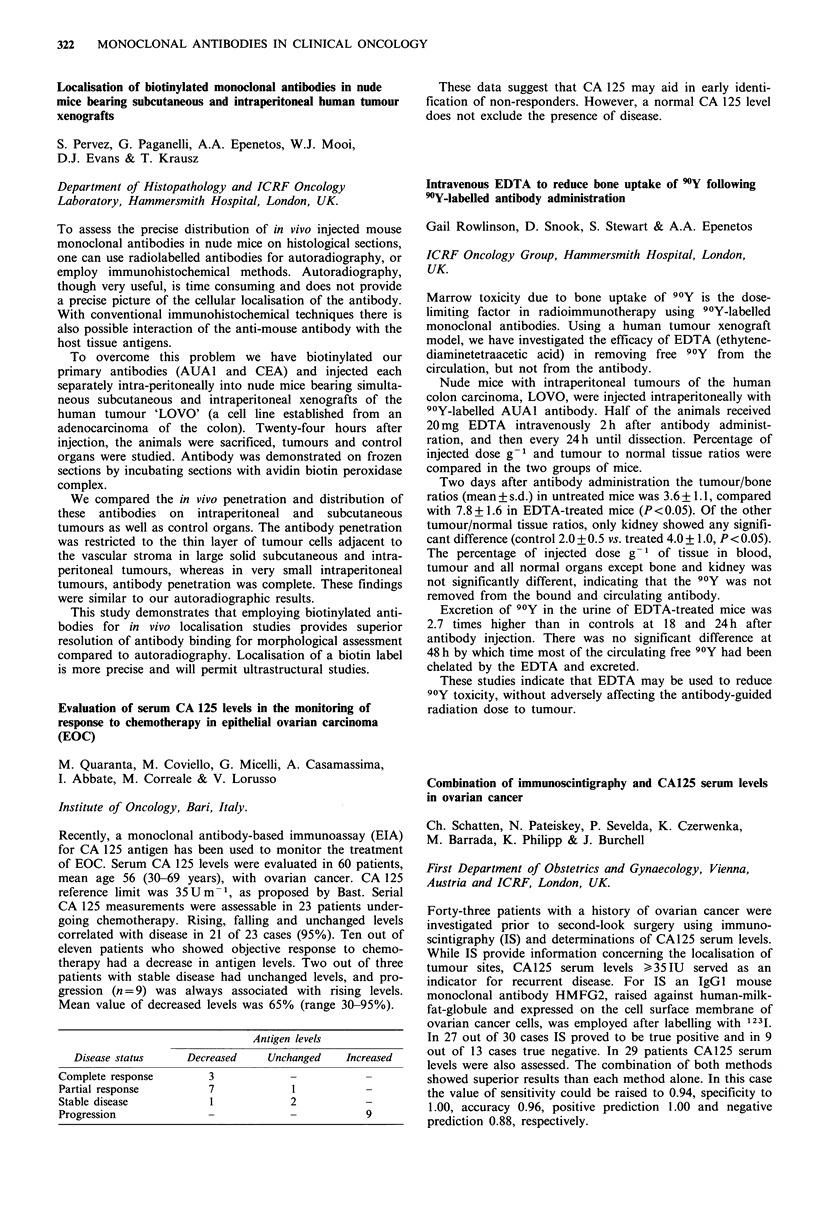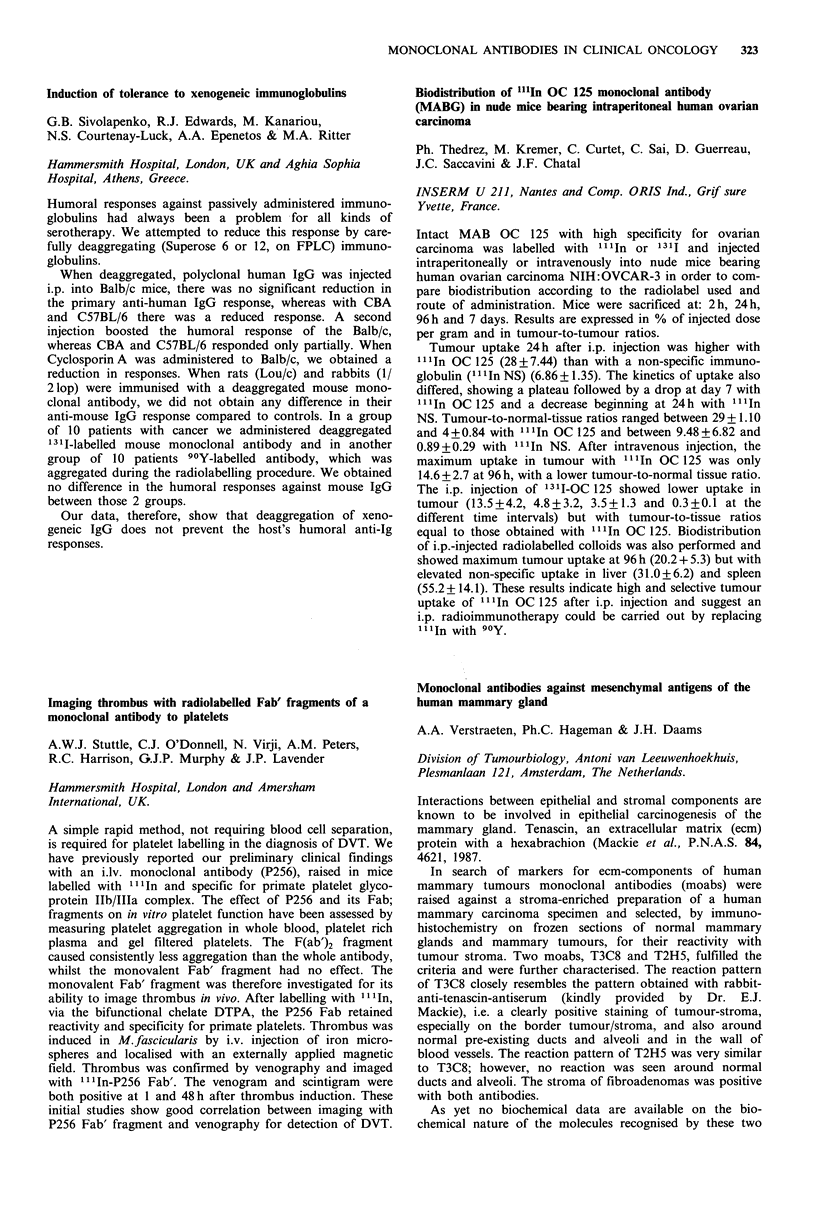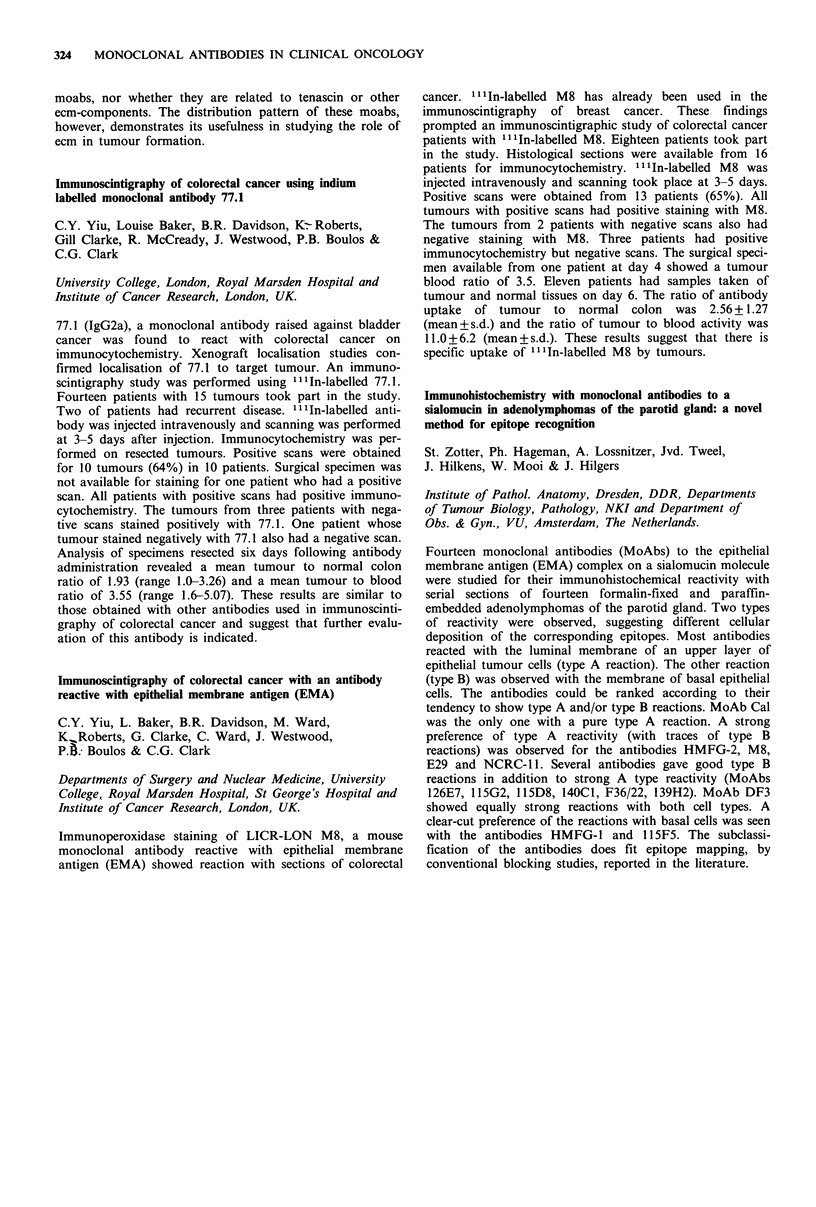# Advances in the applications of monoclonal antibodies in clinical oncology 25/27 may 1988. Abstracts of the proceedings

**Published:** 1989-02

**Authors:** 


*Br. J. Cancer* (1989), **59**, 303–324 © The Macmillan Press Ltd., 1989

### University of London Royal Postgraduate Medical School Symposium on Advances in the Applications of Monoclonal Antibodies in Clinical Oncology

25–27 May 1988

*Held at the Stamp Lecture Theatre, Wolfson Institute, London, UK.*

### Development of immunotoxins for clinical trials

P.E. Thorpe

*Imperial Cancer Research Fund, PO Box 123, Lincoln's Inn Fields, London WC2A 3PX, UK.*

In many *in vitro* test systems, immunotoxins have been shown to kill cancer cells with such outstanding potency and specificity that we are encouraged to think they will provide a new type of chemotherapeutic agent for the treatment of human cancer. However, experiments in tumour-bearing mice have revealed that immunotoxins are subject to a number of problems *in vivo* which reduce their anti-tumour activity. Three of these problems are: (1) ricin A-chain immunotoxins are cleared rapidly from the bloodstream because the oligosaccharides present on the toxin moiety are recognised by the parenchymal and non-parenchymal cells of the liver; (2) commercially available disulphide cross-linking agents used to form A-chain immunotoxins give linkages which are unstable *in vivo*; (3) free antibody is often present as a contaminant in immunotoxin preparations and competes for target antigens. The anti-tumour activity of ricin A-chain immunotoxins is greatly improved by deglycosylating the A-chain to prevent liver recognition, using a new, highly stable cross-linker (SMPT) to form the immunotoxin, and removing the contaminating free antibody by Blue Sepharose chromatography. All but 0.001% of murine T-cell leukaemia cells can be eradicated from tumour-bearing mice by a single intravenous injection of immunotoxin. Immunotoxins of this type can be used to treat patients suffering from B-cell leukaemias and lymphomas.

### Genetic basis of multidrug resistance

V. Ling

*The Ontario Cancer Institute, Toronto, Canada.*

Studies of multidrug-resistant (MDR) mutants have resulted in new insights into anticancer drug transport in mammalian cells. The simultaneous resistance to a wide variety of unrelated compounds in MDR cells appears to be mediated by an increase in a 170,000 daltons plasma membrane glycoprotein (Pgp). Pgp appears to be encoded by a small family of closely related genes. However, gene transfection studies indicate that transfer of a single member of the Pgp genes is sufficient to mediate multidrug resistance. Analysis of Pgp cDNAs revealed that Pgp has remarkable structural homology to a number of bacterial transport proteins, and that it has features (ATP-binding and pore-forming domains) of a membrane pump protein. Thus it is envisioned that a variety of compounds (including many anticancer drugs) are exported from mammalian cells via this molecular pump mechanism.

Despite this understanding of Pgp function, many basic questions remain unanswered. These include: (1) what mechanism(s) regulates Pgp expression? (2) what structural features of Pgp are important for pump activity and for recognising compounds that are exported? (3) is there a normal substitute for Pgp, and what role does Pgp play in normal cells? (4) what role is played by the different Pgp gene members? (5) does the Pgp mechanism of drug resistance play a role in the clinical response of patients to chemotherapy?

### *In vitro* and *in vivo* activities of a mouse-human chimaeric B72.3 antibody

M. Bodmer, N. Whittle, J. Adair, C. Lloyd & J. Schlom

*Department of Molecular Immunology, Celltech Ltd, Slough, UK and NCI, NIH, USA.*

B72.3 is a mouse IgG1 antibody which recognises a tumour associated antigen, TAG-72, found on a variety of human carcinomas, including colon, breast and ovarian. Chimaeric immunoglobulin genes with the B72.3 specificity have been constructed by joining the mouse variable regions from cDNA clones to human genomic gamma 4 and kappa constant regions. The chimaeric immunoglobulin genes were placed under the control of a strong viral promoter, and transfected into COS-1 cells. SDS-PAGE analysis of the $^{35}S$-labelled products demonstrated that the transiently expressed antibodies were correctly synthesised, assembled and secreted. The specific binding characteristics of the parent B72.3 antibody were retained by the chimaeric antibody in an antigen-based ELISA. This system generated sufficiently high transient expression of the chimaeric antibody molecules to allow rapid physical and immunological characterisation of the engineered gene products. Stable cell lines expressing chimaeric antibody were made in CHO cells and purified IgG has been used in quantitative studies. Competition binding assays and tissue staining confirm the COS cell data on activity and specificity. The localisation *in vivo* to LS174T xenografts in nude mice is equivalent to the parent B72.3 antibody.

Course organiser: A.A. Epenetos.
Course secretary: R. Parks.
Both of the Imperial Cancer Research Fund Oncology Group, Department of Clinical Oncology, Hammersmith Hospital, Du Cane Road, London, UK.
Supported by the Imperial Cancer Research Fund.

### Engineering antibody affinity and specificity

D.M. Webster, J.C. Cheetham, S. Roberts, R. Griest & A.R. Rees

*University of Oxford, Laboratory of Molecular Biophysics, The Rex Richards Bldg, South Parks Road, Oxford OX1 3QU, UK.*

In order to understand the way in which antibodies recognise and bind to antigenic surfaces, detailed information about the interactions between antibody and its epitope must be defined. It is now possible to explore the molecular basis of antibody affinity and specificity by combining the techniques of molecular modelling using computer graphics, energy calculations and protein engineering using site directed mutagenesis.

A panel of five monoclonal antibodies (mAb) (gloops 1–5) are used in our laboratory to study antibody:protein antigen interactions. These antibodies were raised against the isolated 'loop' peptide, residues 57–83 of hen egg-white lysozymes (HEWL) and selected by cross-reactivity with native lysozyme. The antibodies are classified into three groups which recognise dissimilar but overlapping epitopes. In addition we are examining antibody:DNA/RNA antigen interactions with mAbs derived from systemic lupus erythematosus prone mice.

Our studies have shown that we are able to specifically engineer antibodies with altered affinities from the native protein. The topographical surface of the anti-lysozyme antibodies can be altered by a small number of changes, resulting in effects on antibody binding, ranging from complete abolition through to increased affinity for antigen. From these studies we hope to establish the rules governing antibody:antigen interactions and that an understanding of such interactions will enable us to design antibodies of clinical and industrial importance.

### Structural analysis of high affinity monoclonal antibodies in the secondary response

Deborah J. Allen, A. Cumano & K. Rajewski

*Imperial Cancer Research Fund, Clare Hall Laboratories, South Mimms, UK and Institut für Genetik der Universitat Zu Koln, Koln, W. Germany.*

The primary immune response of C57Bl/6 mice to the hapten NP (4-hydroxy-3-nitrophenylacetyl) is characterised by light chain-bearing antibodies of germline $V_h$ gene sequence. Upon secondary challenge with the hapten, antibodies of increased affinity are observed which are extensively mutated throughout their V regions. Amongst the mutations observed, a tryptophan to leucine exchange (TCG–TTG) at position 33 of the heavy chain is repeatedly observed in independently isolated hybridomas. We have used site-specific mutagenesis to introduce this single exchange into a primary response antibody and show that the hapten-binding affinity is increased by up to 10-fold. We have also shown that restoring a tryptophan residue at position 33 of a secondary response antibody (3B44) causes a 10-fold decrease in affinity, implying that all other somatic mutations in 3B44 were irrelevant to a selection of improved hapten binding *in vivo*. However, in rare cases where the trp-leu exchange is not observed in the secondary response, we have shown, by recombinant antibody construction, that structures in the third complementary-determining region may influence selection of other mutations in the $V_H$ region which lead to increased affinity.

### Therapy with hybrid antibodies

M. Clark & H. Waldmann

*Division of Immunology, Department of Pathology, University of Cambridge, Tennis Court Road, Cambridge CB2 1QP, UK.*

The cell fusion of two Ig producing cell lines results in the codominant expression of all of the parental cell derived Ig chains. For a cell making two Ig heavy chains and two Ig light chains a random association of all chains results in ten possible configurations of secreted antibody species. The interest in these antibodies for therapy stems from the biological properties of different species within the complex mixture. In addition to the properties which depend upon the interaction of different Ig isotypes with human effector mechanisms there are unique properties which result from the different binding specificities for antigen. For example, certain species of antibody are monovalent for binding to a given antigen. Such monovalent antibodies with specificities for modulating cell surface antigens seem to be more efficient than the equivalent bivalent antibodies at lysing target cells with complement. Monovalent CD3 antibody is currently being used therapeutically. Another of the components of the mixture has dual specificity for the two different antigens recognised by the parental antibody types. These bi-specific antibodies are capable of targeting drugs or toxins to cells expressing appropriate cell surface antigens. Also of interest is the ability of these bi-specific antibodies to induce very potent tumour cell killing by activated T-cell blasts when the bi-specific antibody is used to cross-link a component of the T-cell receptor complex and a suitable cell surface antigen on the target cell.

### Radioimmunoimaging – a critical review

G.L. DeNardo, S.J. DeNardo, D.J. Macey, C.F. Meares & S.V. Deshpande

*University of California, Davis Medical Center, Sacramento, CA, USA.*

Calculations based upon cell and antigen densities and scintillation camera sensitivities suggest that radioimmunoimaging (RII) has the potential in theory to detect cancers smaller than currently possible by existing imaging techniques and prior to likelihood of metastasis. While clinical trials of RII have not been extensive thus far, they have provided results far short of that theoretically possible. Detection of 60–90% of lesions and lesions not detectable by other methods has been reported for several different antibody-radionuclide systems, but lesions with diameters less than 1–2 cm have often been missed. Many factors degrade the imaging potential of RII. Some of these are amenable to solution through improved radionuclides, radiochemistry, antibodies and so forth. More challenging obstacles relate to delivery of the radionuclide to the tumour. Creativity has already led to several proposals for enhanced delivery. These include the use of radiation, biological response modifiers, antibody fragments and hybrid antibodies. It seems wiser at this stage of development to ask the right questions rather than make predictions for the future. The following are among the more interesting questions: can the difference between tumour and tissue vessels be used to enhance tumour uptake; why do some patients develop HAMA; why are some antibodies recognised as different in the species of origin as well as other species; do affinity and avidity influence tumour uptake *in vivo*?

**Dosimetry and suppression of the human antimouse response in iodine-131 labelled antibody therapy of cancer**

R.H.J. Begent

*Cancer Research Campaign Laboratories, Department of Medical Oncology, Charing Cross Hospital, Fulham Palace Road, London W6, UK.*

The techniques of molecular biology and modern immunology give us many molecules which have potential for targeting to cancer. Only quantitative clinical study can identify those which will be of value to patients. Methods for quantitative clinical testing are now being developed with radiolabelled antibodies. Three-dimensional (SPET) gamma-camera imaging is necessary for accurate radiation dose measurements and these must be done from the day of administration for 2–3 weeks. Means of avoiding the inaccuracies inherent in the method have been developed. With this system great variation in the efficiency in antibody localisation has been shown and some prediction of response is possible in patients with colorectal cancer treated with iodine$^{131}$-labelled antibody to CEA. It is also evident that the best therapeutic ratio (11:1) occurs after the first two days, especially if second antibody is given. This favours the use of dual phase system for targeted therapy. Even so it is likely that multiple dose therapy will be needed and prevention of the human antimouse response will be necessary. We have shown that cyclosporin A can delay and reduce the intensity of human antimouse antibody production so that up to four times as many doses of antibody therapy can be given in man. This ability to give repeated therapy and to measure the efficiency of targeting provides the means to select patients for effective anti-tumour therapy.

**Advances in immunohistology**

Eadie Heyderman & A.M.R. Haines

*Department of Histopathology, UMDS, St Thomas' Hospital, London SE1 7EH, UK.*

The majority of histopathological diagnoses are made on the basis of routine H&E preparations, supplemented by special histological techniques such as PAS, reticulin, and Masson–Fontana stains. In the primary care hospital about 3–10% of cases require further immunohistochemical investigations for diagnosis, and/or more precise classification, as for example in lymphomas. There are problems associated with the interpretation of immunohistology, since the technique can only demonstrate stored antigen, and cannot distinguish between synthesised and phagocytosed material. Cells which export their product rapidly may appear negative. Notwithstanding these problems, which may be approached by *in situ* hybridisation of mRNA, useful additional information may be obtained. With the advent of more effective and potentially toxic therapy, definitive diagnosis has become even more important. In addition the possibility of antibody-directed therapy, using conjugates of specific antibodies and various toxins, requires mapping of the distribution of the appropriate antigen, to avoid damage to normal tissues expressing the same epitope as the tumour. The most specific epithelial markers presently available include those directed against prostatic acid phosphatase and against thyroglobulin, although the epitopes recognised by these have been localised on occasional non-prostatic and non-thyroid tissues. Taken together with the H&E morphology other less specific markers may still give important diagnostic information.

**The use of antibodies to study oncogenes, growth factors and their receptors**

W.J. Gullick

*ICRF Oncology Group, Royal Postgraduate Medical School, Hammersmith Hospital, London, UK.*

One of the features that distinguishes tumour cells from normal cells is the ability of the former to grow rapidly in low concentrations of serum. Investigations of this observation at the molecular level have revealed that molecules normally involved in growth control are frequently aberrantly expressed by tumour cells. Many of the genes encoding such molecules can be activated by mutation to forms capable of transforming cells. These genes, termed oncogenes, encode growth factors, growth factor receptors and molecules in the cytoplasm and cell nucleus involved in transduction of growth regulatory signals.

We have investigated the production of growth factors and their receptors in a variety of primary human tumour biopsies. The epidermal growth factor receptors and the structurally related c-erbB-2 protein have been found to be frequently over-expressed in a variety of human carcinomas as assessed by immunohistological staining and Western blotting. The number of gene copies of both these molecules are also often increased in tumours.

We have examined the role of these changes in the instigation and maintenance of the transformed phenotype as well as their value in prediction of tumour behaviour.

**Structure and epitope mapping of an immunogenic region of a human polymorphic epithelial mucin**

Joyce Taylor-Papadimitriou, Sandra Gendler, Joy Burchell, J. Rothbard & T. Duhig

*Imperial Cancer Research Fund, Lincoln's Inn Fields, London, UK.*

The human epithelial mucins are very immunogenic in the rodent, with the result that many antibodies selected for epithelial or carcinoma specificity are directed to these components. It is not yet clear how many genes there are which code for the different mucines, and whether more than one of these genes is expressed by a single cell type. Recently however we have obtained partial cDNA clones for the human mammary mucin which have allowed us to predict the sequence for part of the core protein of this mucin and to look for its expression in a variety of cell types. The gene shows a polymorphism which is due to the presence of varying numbers of a 60bp tandem repeat element. Southern blots of restriction enzyme digests show that only one gene with this structure is present in the human genome (two alleles) indicating that other mucin genes do not show a strong homology, at least in this region. The mucin probe cross-reacts with digests of primate DNA, but not with rodent, thus explaining the immunogenicity of the mammary mucin in mice. A synthetic peptide corresponding to the a.a. sequence predicted from one open reading frame of the tandem repeat reacts with antibodies HMFG-1, HMFG-2 and SM-3, each of which binds to a different epitope. The identification of epitopes which are preferentially expressed on carcinomas should allow (1) a directed approach to the production of more tumour specific antibodies and (2) a study of the differences in the processing of the mucin seen in carcinomas.

### Uptake of radiolabel in rat liver cells after administration of radiolabelled B72.3 and its $F(AB')_2$ fragments

H. Sands, P.L. Jones, B.A. Brown & T. Nason

*Medical Products Department, E.I. duPont & Co., N. Billerica, MA, USA.*

We have previously reported that in rats injected with intact murine anti-human tumour antibody, B72.3, labelled with either $^{125}I(I)$ or $^{111}In(In)$, the liver uptake of radiolabel is predominantly due to accumulation by the liver parenchymal cells. To determine the effect of removal of the Fc portion on liver cell uptake we have measured the uptake of radiolabel by rat liver cells of animals injected i.v. with either intact B72.3 and its F(ab')2 labelled with either I or In. One hour after administration, liver cells, obtained by collagenase perfusion, were separated into parenchymal (PC) and non-parenchymal (NPC) fractions using Percoll gradients. 77.9% and 80.8% of the label recovered was PC-associated when I- or In-labelled B72.3 was administered, while after I- and In-F(ab'2) administration, 96% and 94.6% of the label was recovered in the PC fractions. The absolute uptake of In-F(ab')2 was similar to that of I-F(ab')2 but was 54% of that seen with the IgG. To determine whether the lower absolute liver uptake of F(ab')2 was due to the absence of Fc binding or was a result of more rapid blood clearance, the uptake of B72.3 and its F(ab')2 labelled with I or In was measured in the perfused rat liver. When compared with data obtained from livers of rats injected with labelled antibodies, the uptake of I- and In-B72.3 in the perfused liver was reduced by 57.2% and 92.3%, respectively, by removal of Fc. Thus Fc binding is a major component contributing to liver uptake of IgG. When the liver uptake of B72.3 and its F(ab')2 labelled with $^{99}Tc^m$ using rabbit metallothionein as the radionuclide chelator was studied, >84% of recovered $^{99}Tc^m$ was in the PC fraction. In summary, PC are the major liver cell of radionuclide accretion regardless of either the specific radionuclide or whether the label is attached to IgG or F(ab')2.

### Immunogold and immunogold/silver in the ultrastructural localisation of target molecules identified by monoclonal antibodies

Rosemonde Mandeville & M.G. Zelecowska

*Immunological Research Center, Institut Armand-Frappier, Laval-des-Rapides, Quebec, Canada H7N 4Z3.*

The use of colloidal gold and colloidal gold followed by silver enhancement are two highly sensitive and accurate techniques for the visualisation of target molecules identified by monoclonal antibodies (MAbs). Gold probes were particularly useful when used at two different particle sizes (5 and 20 nm in diameter) in a double labelling method for the identification of two antigens on the same grid. We have also combined gold and gold/silver staining in a double staining technique for the simultaneous localization of breast cancer-associated antigens using BCD-E8 and BCD-F9 Mabs. These techniques have also been used to demonstrate the internalisation of the gold-labelled BCD-F9 MAb in MCF-7 cells by warming for 30 min at 37°C. We conclude that colloidal gold and gold/silver markers constitute valuable and versatile tools for the ultrastructural localisation and quantitation of target molecules identified by MAbs and that further development of these techniques may have an important impact on the development and use of MAbs in oncology.

### The presence of multiple tumour-associated epitopes on an immunopurified antigen and its use to generate further monoclonal antibodies

A.B. Griffiths & Joyce Taylor-Papadimitriou

*Imperial Cancer Research Fund, London, UK.*

The monoclonal antibody HMFG2 is well characterised and recognises a determinant on high molecular weight glycoproteins present in epithelial cells and associated with malignancy, notably ovary and breast. In comparative immunoblots we have shown that many antibodies raised and selected for their reactivity with carcinomas also react with glycoproteins similar to those recognised by HMFG2. We have immunopurified material bearing the HMFG2 component on an HMFG2 affinity column from membrane preparations of the breast cell lines T47D and MCF7 and demonstrated the cross-reactivity of this immunopurified material with other antibodies in a solid phase ELISA assay. This confirms that this material bears many tumour associated determinants, defined by monoclonal antibodies, and may be important in the study of the malignant phenotype. Western blots and silver staining of the PAGE separated immunopurified material demonstrate the presence of the high molecular weight components. This material has been used to generate further monoclonal antibodies. These include two antibodies which, on Western blots of PAGE separated T47D and MCF7 cells, recognise, in addition to the high molecular weight material, a band at 68 K identical to that seen in the immunopurified material used as antigen. This suggests that the 68 K material recognised by these antibodies may share a function as well as immunological relationship with the high molecular weight material on which many cancer related determinants are expressed. Other antibodies produced recognised a large proportion of breast cancers and may be useful for immunolocalisation.

### Enzyme immunoassay detection of ovarian cancer-associated sebaceous gland antigen (SGA) on biological fluids

G.T. Layton, M.J. Baily, P.L. Devine, G.W. Birrel & J. Golder

*Medical Innovations Ltd., 11 Technology Drive, Labrador, Queensland 4215, Australia.*

The monoclonal IgM antibody OM-1 has previously been shown by immunohistology to react with primary and metastatic ovarian serous cystadenocarcinomas, endometroid carcinomas and with ovarian tumour cell lines. The antigen was also found in sebaceous glands in normal skin and was, thereore, termed sebaceous gland antigen (SGA). In order to investigate the presence, distribution and possible significance of SGA molecules in serum and other biological fluids we have developed and sandwich enzyme immunoassay. OM-1 was purified from ascites and an aliquot was biotinylated. The assay procedure involves firstly coating microtitre plate wells with OM-1 antibody. Then the test samples, OM-1 biotin, streptavidin-horseradish peroxidase conjugate and finally substrate are sequentially incubated in the coated wells, with washes between each stage to remove unbound material. Using this method, SGA was detected in the culture supernatants of ovarian tumour cell lines and in sera and ascites from patients with ovarian cancer. SGA could also be detected in a human milk fat globule membrane preparation. A semi-quantitative assay has now been established to evaluate the diagnostic and therapeutic monitoring potential of SGA measurement in ovarian cancer.

**Preoperative discrimination between benign and malignant ovarian tumours using a combination of CA125 and CA15.3 serum assays**

C. Yedema, L. Massuger, J. Hilgers, L. Poels, C. Schijf, R. Verheijen, C. Thomas & P. Kenemans

*Department Ob/Gyn, Radboud Hospital, Nijmegen and Department Ob/Gyn, Free University Hospital, Amsterdam, The Netherlands.*

Most ovarian cancer patients have elevated CA125 levels in pretreatment serum. A number of patients with benign ovarian neoplasms, are also found to have elevated CA125 serum levels. Therefore, if CA125 levels are measured alone, an overlap between both groups is observed. The present study was initiated to achieve a better discrimination between both groups mentioned, using a combination of two tumour marker serum assays CA125 and CA15.3. In pre-operative sera of 38 well staged ovarian cancer patients and 32 patients with benign ovarian neoplasms both CA125 and CA125 ($>35\,U\,ml^{-1}$) were observed in 71% and elevation of CA15.3 ($>30\,U\,ml^{-1}$) in 50%. Within the group of patients with benign ovarian neoplasms, elevation of CA125 and CA15.3 was observed in 25% and 9% respectively. Of these ovarian cancer patients with normal CA125 serum levels, 10 out of 11 had stage I disease and a borderline or a mucinous carcinoma. In the entire group of 70 patients, CA125 serum levels were elevated in 35 patients, of which 27 had ovarian cancer and 8 a benign ovarian tumour. Out of 8 patients with false positive CA125 elevation only one had an elevated CA15.3 level, whereas in 27 correct positive patients 19 had elevated CA15.3 levels.

In cases of simultaneous elevation of both tumour markers (20 patients) 95% had ovarian cancer. If for CA125 a cut-off level of $65\,U\,ml^{-1}$ was used, all patients with both tumour markers elevated (19 patients) were found to have an ovarian malignancy. Determination of a panel of tumour markers seem to be useful in the preoperative screening of ovarian tumours.

**Some aspects of antibody labelling with metallic radionuclides**

H. Maecke, T. Kaden, A. Riesen, W. Ritter & M. Studer

*University Hospital Basel, Departments of Nuclear Medicine and Chemistry, University of Basel, Switzerland.*

Bifunctional ligands with the ability to bind metallic radionuclides and to be covalently linked to biomolecules are of interest due to the need of versatile methods for the labelling of antibodies. We synthesised functionalised macrocycles and examined their suitability in the labelling of antibodies with $^{64}Cu$, $^{57}Co$ ($^{55}Co$), $^{111}In$ and $^{90}Y$. Monofunctionalised macrocycles proved to be appropriate for $Cu^{2+}$ and $Co^{2+}(Co^{3+})$. The complexes are extremely stable and no loss of cobalt or copper was found even in acidic solutions for days.

Moreover new ligands were synthesised designed to complex $^{111}In^{3+}$ and $^{90}Y^{3+}$. Convenient starting materials to bifunctional ligands 1,4,8,11-tetraazacyclotetradecane-*N,N′N″*-triacetic acid (14-ane$N_4$triac) were synthesised. Alkylation at the secondary amine results in bifunctional compounds. We studied aspects of basic $In^{3+}$-coordination chemistry in relation to antibody labelling as well as kinetic and thermodynamic data. Some of the complexes were fully characterised by X-ray crystallography. Interestingly In(14-ane$N_4$.4$H_2O$) complex is octa-co-ordinate. The dtpa-ligand binds through the three bitrogen donors and five deprotonated carboxylate groups.

**A new monoclonal antibody (MOv18) against ovarian cancer for imaging and therapy**

F. Crippa, M. Gasparini, E. Seregni, S. Canevari, M. Ripamonti, F. Landoni, R.V. Fontanelli, G.A. Scassellati & G.L. Buraggi

*National Cancer Institute, Milan, Ospedale S. Gerardo, Monza and Sorin Biomedica, Italy.*

A new monoclonal antibody (MOv18) against human ovarian cancer was labelled with $^{131}I$ using the iodogen method and prepared for human administration by Sorin Biomedica, Saluggia. Clinical trials with radiolabelled MOv18 are in progress to evaluate its possible applications in tumour detection and therapy. At present, 20 patients with advanced ovarian carcinomas received intravenous or intraperitoneal MOv18 antibody (mean activity injected 86.81 MBq, mean quantity of monoclonal antibody 0.425 mg), and its biodistribution and tumour localisation were evaluated by sequential immunoscintigraphy during weeks 1–2 after administration. All the patients were subjected to a surgical second look after immunoscintigraphy. Our preliminary results demonstrated that MOv18 is a suitable reagent for *in vivo* (immunoscintigraphy of ovarian carcinoma and potential radioimmunotherapy.

**Successful imaging of metastatic tumours using labelled monoclonal antibodies: A prelude to therapy**

P.G. Abrams, R.W. Schroff, M.F. Fer, D. Salk, D.S. Wilbur, J. Sinkule, J.F. Eary & W.B. Nelp

*NeoRx Corporation and the Division of Nuclear Medicine, University of Washington, Seattle, USA.*

Therapeutic applications of monoclonal antibodies are dependent at least on their targetting to tumours without significant concurrent localisation in normal organs. Thus, successful imaging strategies serve as a safe, rapidly developed prelude to therapy. To date, nearly 200 patients with melanoma, lung and colon cancer have been successfully imaged using Fab fragments of NR-M1-05, NR-Lu-10 and NR-Ce-01 antibodies all linked to $^{99}Tc^m$ by a stable chelating system. Toxicity, allergic in nature, was rare (1.5%), readily reversible and never prevented completion of the study. Images were obtained in 6–20 h. Previously known and occult metastases were detected in liver, bone, skin, brain, lymph node, adrenal, ovary, spleen and lung, the latter having a lower detection rate for reasons as yet unexplained. Accumulation of the labelled antibody was observed in thyroid (cross-reactivity) and occasionally in areas of osteoarthritis positive on bone scan, and infrequently in inflammatory lesions. Excretion of the label via the renal and hepatobiliary routes complicated image interpretation. Results of imaging assisted in patient management. We conclude that $^{99}Tc^m$ may be stably chelated to antibody fragments. The resulting immunoconjugates provide useful staging information in a single diagnostic scan. Elimination of hepatobiliary excretion will improve image interpretation. We have also shown that stable iodine and rhenium chelated antibodies, as well as doxorubicin, trichothecene conjugates, can target tumours in nude mice equal to antibody alone.

### The use of macrocyclic ligands in radioimmunoconjugates

M.A.W. Eaton, D. Parker & A. Harrison

*Celltech Group, plc, Department of Chemistry, University of Durham and MRC Radiobiology Unit, UK.*

The essence of effective and efficient radioimmunodiagnosis and therapy is to ensure that the radionuclide remains attached to the antibody *in vivo*. None of the commonly used acyclic chelates (EDTA, DTPA-based) are really adequate for binding metallic isotopes because of the facility of metal dissociation. Such problems may be obviated with macrocyclic ligands (provided that they are judiciously designed) because acid-promoted dissociation is much slower. New methods have been developed for the selective attachment of these macrocycles to thiol residues on an antibody. Conditions for radiolabelling the macrocycle–antibody conjugate have been established, and animal data for $^{99}Tc^{m}$ and $^{64}Cu$ labelled antibody are encouraging. These results have clinical implications and could lead to improved protocols for $^{186}Re$ and $^{67}Cu$ radioimmunotherapy.

### Polyazamacrocyclic chelating agents for radioimmunoimaging and radioimmunotherapy

O.A. Gansow, M.W. Brechbiel, K. Kumar, S. Mirzadeh, T. McMurry & M. Magerstadt

*National Institutes of Health, Bethesda, MD 20892, USA.*

Polyazamacrocyclic polymethylenecarboxylate chelating agents exhibit the highest thermodynamic stability constants known for the radionuclides $^{90}Y$, $^{203}Pb$, $^{212}Bi$ of potential use in imaging and radioimmunotherapy. Yet no studies of the DOTA complexes with these isotopes or of the synthesis of a bifunctional DOTA have been published.

We report that complexes of the above named radionuclides with DOTA have been synthesised and their kinetics of isotope exchange and dissociated in aqueous media examined. All complexes are kinetically inert from pH 3 to 9.

In view of the likely importance of $^{212}Pb$ as a carrier of alpha emitter $^{212}Bi$ for radioimmunotherapy, we have carefully examined the retention of the $^{212}Bi$ in DOTA when produced by radiodecay of the parent $^{212}Pb$ and are able to report substantial retention of the daughter radionuclide. Syntheses for 1-(*p*-isothiocyanatobenzyl)DOTA have been achieved.

### Tumour localisation studies with streptavidin and biotin

D.J. Hnatowich, G. Rowlinson, M. Rusckowski, D. Snook & A.A. Epenetos

*University of Massachusetts, USA and Hammersmith Hospital, London, UK.*

Using a human tumour xenograft model, we are investigating the use of streptavidin-conjugated anti-tumour antibody HMFG1 (S-G1) administered prior to the administration of radiolabelled biotin as a means of improving tumour targeting. Nude mice implanted subcutaneously with HEP-2 tumour, were administered i.v. with 9 $\mu$g S-G1 and, at 1, 4 and 7 days thereafter, were administered with $^{111}In$-biotin. Biodistribution was compared with that obtained in the conventional manner with $^{111}In$-HMFG1. Animals were killed and dissected 2 h after administration of biotin at either 0.1, 1, 10 or 100 $\mu$g per animal. Radioactivity levels in normal tissues were substantially reduced at all time points with respect to the conventional labelling procedure. These levels were also reduced in tumour, the tumour to normal tissue ratios (with the exception of kidneys) were improved up to four-fold.

These studies demonstrate the potential of this approach for tumour imaging, at earlier times after administration than with the conventionally labelled antibody.

### *In vivo* tumour localisation by means of biotinylated monoclonal antibodies and radioactive streptavidin: a novel approach to cancer targeting

G. Paganelli, G. Rowlinson, S. Pervez, G. Deleide, F. Chiolenio, M. Malcovati, A.G. Siccardi & A.A. Epenetos

*M. Buffalini Hospital, Cesena, Italy and Hammersmith Hospital, London, UK.*

Nude mice bearing intraperitoneal human colon carcinoma tumours were injected with biotinylated monoclonal antibody AUA1, followed 24 h later by radioiodinated streptavidin as a control study. A direct method of peroxidase staining using peroxidase-conjugated streptavidin showed the *in vivo* presence and distribution on tumour of biotinylated MoAbs. Autoradiography was also performed as a comparison study to demonstrate targeting of radioactive streptavidin to biotinylated antibody. Our results show that this two-step approach: (1) increased the percentage of radioactivity uptake by tumour (24% *vs.* 6%); (2) improved the target to non-target ratio (improvement index greater than 6.5); (3) has advantages over a single step approach using radiolabelled antibody *in vivo*.

### Sensitivity, toxicity and immunogenicity of human monoclonal antibodies in imaging patients with colon cancer

R.P. McCabe, R.G. Steis, J.A. Carrasquillo, J.C. Reynolds, M.V. Haspel, E.M. Paris, M.M. Kopciensky & M.G. Hanna, Jnr.

*Organon Teknika Corporation, Bionetics Research Institute, NCI and NIH, USA.*

Positive tumour images were obtained in 20 out of 24 patients with colon cancer receiving $^{131}I$-labelled human monoclonal antibodies 16-88 or 28A32. Lesions as small as 1.5 cm were detected. Limited antibody related toxicity (urticaria) was seen in two patients. This was not dose related. No evidence of toxicity was seen with 16-88. Sera were tested for anti-antibody activity up to four weeks after receiving up to 200 mg human monoclonal antibody for four consecutive weeks. No anti-16-88 activity was seen in any patient. Anti-28A32 activity was seen in pre-treatment sera from two patients with positive skin tests who did not enter the study, from the two patients who developed urticarial rashes after infusion of 28A32 and from one of two patients whose tumour was not detected by $^{131}I$-28A32. Those patients who received 28A32 infusion exhibited only a four-fold increase in anti-28A32 titre over the 2-month observation period. At present it is not known whether the pre-existing anti-antibody activity is anti-allotypic or anti-idiotypic. However, evidence suggests that pre-existing antibody to 28A32 may be a selection factor in identifying the most appropriate human monoclonal antibody for use in patients, and that a panel of human monoclonal antibodies is needed to provide the most appropriate antibody for the greatest number of patients.

### Comparison of the specificity of monoclonal antibodies to malignant gliomas

B.A. Watkins, Pat Carter, F. Scaravilli, L.W. Duchen & D.G.T. Thomas

*Institute of Neurology, Queen Square, London WC1N 3BG, UK.*

Recent developments in the differential diagnosis of the small cell cancers of childhood have centered on the use of monoclonal antibodies to identify cell-type specific antigens in specific types of neoplasm. In addition, radiolabelled monoclonal antibodies have proven useful in tumour imaging and in the treatment of diffuse neoplastic meningitis.

In an attempt to extend the use of monoclonal antibodies into differential glioma diagnosis, antibodies were raised against three malignant gliomas, using standard techniques. A total of 281 hybridoma clones were raised and tested for reactivity against the glioma used for the initial immunisation. Six antibodies were selected for further study on the basis of their strong anti-glioma reactivity with non-neoplastic brain tissue.

These antibodies were tested against a large panel of glial and non-glial brain tumours, and each showed a highly characteristic staining pattern. The patterns of reactivity seen in sections stained with these antibodies suggest that each antibody stains specific groups of cells within glial tumours. The specificity shown by these antibodies for glioma tissue as opposed to normal brain, and their apparent specificity for particular sub-sets of glioma cells suggest that these antibodies may prove useful in the diagnosis, imaging and therapy of malignant gliomas.

### Radioimmunoscintigraphy of high-grade brain gliomas

D.V. Skarlos, J. Malamitisi, G. Augustatos, N. Demakopoulos, A. Varvarigou, T. Giannakakis, P. Economidis, C. Kotoulidis & A. Epenetos

*3rd Med. Onc. Department of Ag. Anargiri Cancer Hospital, Nucl. Department of NIMTS Hospital, Athens and ICRF Onc. Group, Greece.*

Ten patients with relapsed gliomas grade III, IV and a patient with astrocytoma grade I were studied with $^{131}$I-H17E2. Additionally, two patients with glioma III, IV were studied with a non-specific anti-idiotype 2118 MCA. 1.5–2.0 mCi (55–74 MBq) were administered intracarotically (i.c.) or intravenously (i.v.). Immunoscans were taken at 0, 2, 24, 48 and 72 h and kinetics of the MCAs were studied. See table.

$^{131}$I-H17E2 produced positive scans with gliomas grade III, IV but not with astrocytomas. These findings correlate with immunohistochemical data. Intravenous route of administration is equivalent to i.c. route. $^{131}$I-H17E2 appears promising for immunotherapy.

| | | | Tum./NT | | | |
|---|---|---|---|---|---|---|
| | | | Immunosc. | | ratio | |
| Patient no. | CT | Route of adm | 48 h | 72 h | 48 h | 72 h |
| 1 | + | IC | + | + | 2.25 | 2.34 |
| 2 | + | IC | + | + | 1.43 | 1.42 |
| 3 | + | IV | + | + | 1.79 | 1.87 |
| 4 | + | IV | + | + | 2.20 | 2.45 |
| 5 | + | IV | + | + | 1.49 | 2.21 |
| 6 | + | IV | + | + | 1.37 | 1.41 |
| 7 | + | IV | + | + | 1.50 | 1.72 |
| 8 | + | IV | n.t. | + | n.t. | 1.74 |
| 9 | + | IV | − | ± | 1.19 | 1.25 |
| 10 | + | IV | − | − | 1.10 | 1.07 |
| 11 | + | IV | + | + | 1.87 | 1.89 |
| 12 | + | IV | ± | − | 1.23 | 1.09 |
| 13 | + | IV | − | − | 1.04 | 1.09 |

Patient 10 had astrocytoma grade I. Patient 11 received non-specific MCA three weeks after administration of H17E2. Patient 13 received only non-specific MCA.
n.t. = not taken.

### Scintigraphic tumour imaging with biotin-conjugated antibodies and radiolabelled streptavidin

P. Oehr, J. Westermann, U. Germer, A. Bockish & H.J. Biersack

*Institute of Nuclear Medicine, University of Bonn, FRG.*

We used nude rats bearing cytokeratin-positive solid HeLa cell carcinomas on their hind leg. The binding of monoclonal anti-cytokeratin antibodies to the tumour was demonstrated *in vitro* by immunohistochemical staining and *in vivo* by means of radioimmunodetection with $^{125}$I-labelled antibodies. The animals were preinjected i.v. with 'cold' biotin-conjugated anti-cytokeratin antibodies. At a later time $^{125}$I-labelled streptavidin was administered i.v. In another approach the animals received an additional injection with a specific anti-antibody to the previously administered anti-cytokeratin antibody in order to reduce free antibodies in the circulation. In the streptavidin–biotin system the tumour could be localised within 60 min, whereas it took at least three days with the directly labelled antibody. Using the anti-antibody the tumour could be seen 15 min after injection. A limitation of the system was some antibody-independent binding of streptavidin to the tumour as well as to other organs, especially kidney and liver.

### Immunoscintigraphy in breast cancer with $^{123}$I-labelled monoclonal antibodies (MOAbs)

S.E.M. Clarke, C. Twelves, C. Lazarus, A. Girling, S. Allen, S. Mather, J. Taylor-Papadimitriou & I. Fentiman

*Department of Nuclear Medicine, ICRF Breast Unit, Guy's Hospital and ICRF, Lincoln's Inn Fields, London WC2, UK.*

The presence of tumour involved nodes in patients with breast cancer can at present only be demonstrated following surgical resection. We have assessed the uptake of two $^{123}$I-labelled MoAbs, HMFG1 and SM3 in patients undergoing mastectomy with axillary clearance. Fourteen patients have been studied, 4 with HMFG1 injected subcutaneously (s.c.) into the finger webs of both hands, 4 with SM3 injected s.c., and 6 with SM3 injected intravenously (i.v.).

Patients were imaged at 1, 4 and 20 h after injection and blood and urine samples were collected. The resected specimen was imaged, the nodes and tumour then dissected, counted individually and examined histologically.

Imaging showed symmetrical uptake in both axillae following subcutaneous injection, and no tumour uptake was observed after IV injection of SM3. Determination of amount of antibody in modes and tumour showed no significant uptake in tumour or tumour bearing nodes following s.c. or i.v. administration of either antibody. The following table shows the mean percentage injected $^{123}$I MoAb/g of tissue.

| | *Normal nodes* | *Tumour nodes* | *Tumour* | *Normal* |
|---|---|---|---|---|
| Breast | | | | |
| HMFG1 (s.c.) | 0.01 | 0.007 | – | – |
| SM3 (s.c.) | 0.007 | 0.005 | – | – |
| SM3 (i.v.) | 0.004 | 0.003 | 0.005 | 0.005 |

In summary we have demonstrated a valuable model for evaluating the uptake of MoAbs in patients with breast cancer. Preliminary data suggest no specific uptake of either HMFG1 or SM3 in patients with breast cancer.

### Immunoscintigraphy with $^{131}$I-labelled HMFG2 and F (ab′)$_2$ HMFG1 in the pre-operative detection of clinical and subclinical lymph node metastases in breast cancer

A. Athanassiou, D. Pectasides, J. Taylor-Papadimitriou & A. Epenetos

*Anticancer Hospital, Piraeus, Greece and ICRF, London, UK.*

In an attempt preoperatively to detect clinical and subclinical axillary lymph node metastases in breast cancer, we used $^{131}$I-labelled MoAb HMFG2 and HMFG1 F(ab′)$_2$. We studied 10 patients with clinically obvious axillary lymph node disease (group A), 10 patients with clinically negative axilla (group B), using MoAb HMFG2, 5 patients with clinically negative axilla (group C), using MoAb HMFG1 F(ab′)$_2$ and 6 patients with clinically positive axillae (group D) using non-specific MoAb 11.4.1 and 4C4. All patients had clinical diagnosis of breast cancer. Each patient received 1–1.5 mCi as a subcutaneous injection into the webs between the 2nd and 3rd fingers of both hands. The patients were scanned at 24–96 h post-injection. In group A, 7 patients had positive scans and 3 negative. The histology and immunoperoxidase staining confirmed the presence of tumour in the lymph nodes in all patients. In group B there were 4 true positive scans, 4 true negative, 1 false positive and 1 false negative. Lymph node involvement was histologically confirmed in 5 patients. In group C there were 4 true negative scans. In 1 patient the MoAb stopped in the middle of the right arm, due to lymphatic obstruction. The histology confirmed the absence of tumour in the lymph nodes. In group D, there were 3 true negative scans with MoAb 11.4.1 and 3 true negative scans with MoAb 4C4. The presence of tumour was histologically confirmed in all patients.

This preliminary study suggests that this approach can preoperatively detect metastatic tumour in axillary lymph nodes and be of value in the diagnosis and staging of breast cancer.

### A Japanese phase II study of immunoscintigraphy with $^{131}$I anti CEA and anti CA19-9 monoclonal antibodies

T. Inoue, Y. Sasaki, N. Oriuchi, N. Mitsuhashi, D. Tsujino, T. Maehara & Y. Suzuki

*Gunma University and five other Institutes, Japan.*

The purpose of this study was to evaluate the safety and clinical usefulness of immunoscintigraphy with $^{131}$I anti CEA and $^{131}$I anti CA19-9 monoclonal antibody cocktail (IMACIS 1) which was provided by International CIS (France). Thirty-six patients with proven cancer in six hospitals were entered in the phase II study. Images were taken with a gamma camera after injection of IMACIS 1 collecting 300 k counts for each view.

Changes of the heart rates, temperature, blood pressure, respiratory rates and other parameters were obtained. Blood clearance, urinary excretion and HAMA response were also investigated.

Positive scintigrams were obtained in 30/36 (83%) patients and in 39/52 (75%) cancer sites. Blood clearance curves constituted of two exponential components with mean $T_{1/2}$ of 7.5 h and 35.4 h. Fifty per cent of the injected dose was excreted in the urine by the 5th day after injection. No significant changes of the heart rates, temperature, blood pressure, respiratory rates and clinical laboratory data were observed. Significant elevation of HAMA titre was not shown. No adverse reactions were observed in any of the patients.

On the basis of these results, we plan to commence phase III clinical trials in Japan.

### T cell activation by anti-idiotypic antibody: implications for vaccine design

Ann D.M. Rees

*MRC Tuberculosis and Related Infections Unit, Royal Postgraduate Medical School, Hammersmith Hospital, London, UK.*

One prediction of Jerne's network hypothesis is that for each foreign antigenic determinant encountered by the immune system there must be an idiotype that bears the internal image. Anti-idiotypic antibodies have possibilities therefore, as surrogate antigens in vaccine design. In anti-mycobacterial immunity, however, T-cells mediate protection. Thus our studies have been directed to the interaction of an anti-idiotypic antibody (anti-Id TB71) with T-cells reactive to the myobacterial antigen (38 kD) defined by the mouse monoclonal (TB71) used to generate anti-Id TB71. It was found that 38 kD specific human T-cell clones also responded to anti-Id TB71. This suggested that this antibody contained an internal image in a T-cell stimulatory domain of the 38 kD molecule. Furthermore, this responsiveness did not depend on the conformational integrity of the antibody and was MHC restricted. This suggested that the antibody was presented to antigen reactive cells by the same mechanisms as conventional antigen. Anti-Id TB71 may, therefore, stimulate an overlapping T-cell repertoire to the 38 kD antigen. These findings should help in vaccine design.

### Treatment of B-cell lymphoma with monoclonal anti-idiotype (Id) antibodies

R. Miller, Sherri Brown & R. Levy

*Stanford University, Stanford, CA and IDEC Pharmaceuticals Corporation, Mountain View, CA, USA.*

Because of their tumour specificity and intrinsic biological activity, anti-Ids are appealing agents to study for the treatment of B-cell lymphomas. We have tested the clinical activity of anti-Ids alone, in combination with alpha interferon (IFN) and radioconjugated with $^{131}$I. Fourteen patients with advanced refractory B-cell lymphoma were treated with anti-Id alone. One complete response (CR) lasting 6 years, and 7 partial responses (PR) of 1 month to greater than 16 months duration were observed. Treatment with anti-Id in combination with IFN (12 million u m$^{-2}$ three times a week for 8 weeks) resulted in 1 CR (13 months) and 7 PR (duration 2–15 months). In both studies, evidence for tumour escape with Ig+, Id− disease was observed. Patients were treated with up to 12 infusions of anti-Id to total doses of 8.4 g. In most responding patients, tumour regression continued long after anti-Id disappeared from the serum. Host anti-mouse Ig response has been avoided in most patients by increased antibody purity. In order to deal with Id negative tumour escape, we are using both anti-Id in combination with chemotherapy and radiolabelled with $^{131}$I. A patient treated with $^{131}$I-anti-Id achieved a CR lasting 10 months. We conclude that anti-Id antibodies have reproducible objective anti-tumour activity in B-cell lymphoma. Strategies which effectively deal with Id negative lymphoma cells should improve the extent and duration of these responses.

### Lymphoma idiotypes as a therapeutic target

G.T. Stevenson, M.J. Glennie & F.K. Stevenson

*Lymphoma Research Unit, Tenovus Laboratory, General Hospital, Southampton, UK.*

Idiotypic determinants of lymphoma cells on Ig molecules of B-lymphomas or TCR molecules of T are unique differentiation antigens offering a well characterised test-bed for immunological destruction of neoplasms. The infusion of anti-idiotypic antibody in patients with lymphoma has proved a relatively innocuous procedure, but in general has yielded only partial, short-lived remissions of disease. A major problem is that xenogeneic antibody is not well fitted to destroying mammalian cells: complement and cellular effectors (K-cells and phagocytes) are not efficiently recruited, and the target cells present some excellent defence mechanisms such as antigenic modulation. Two broad types of antibody derivative are under development to improve the killing of neoplastic targets. One type relies on recruiting natural effectors, and is exemplified by univalent chimeric antibody. The other relies on delivering an exogenous effector such as a drug or toxin and is exemplified by bispecific anti-Id/anti-saporin $F(ab')_2$ antibody. Both types of derivative can suppress animal lymphoma to the extent that tumour escape occurs largely through the emergence of idiotype-negative mutants. An argument thereby arises for simultaneously attacking two cell surface antigens.

Lymphoma idiotypes also have attractions as vaccines for suppressing resurgence of lymphoma after its therapeutic removal but transplantable animal lymphomas eventually overcome active anti-idiotypic suppression, sometimes via modulation, sometimes via mutations.

### Replacement of a mucin-associated carbohydrate epitope by an internal image anti-paratope MAb (Ab2B)

K. Bosslet, J. Friesen, E.J. Kanzy & H.P. Harthus

*Behring Research Laboratories, Marburg, W. Germany.*

MAb BW 494 binds to a carbohydrate epitope located on a >200 kDa mucin molecule. The epitope is expressed on highly differentiated adenocarcinomas of the pancreas, colon and stomach as well as on mucinous ovarian carcinomas. In a attempt to use the carbohydrate epitope as a tumour specific vaccine we tried to mimic this structure producing (a) MAbs binding to the paratope of MAb BW 494 and (b) MAbs inducing an Ab3 response with a specificity identical to that of MAb BW 494.

Syngeneic Bablb/c mice were immunised with the Fab′ fragment of MAb BW 494. Six anti-paratope MAbs which were able to replace the mucin antigen were established, purified and coupled via GMBS to KLH. One out of these 6 MAbs, MAb BW 705, was able to induce in syngeneic (Balb/c mice) and xenogeneic animals (rabbits) an antiserum (Ab3) binding to pancreatic tumour cell associated mucin and inhibiting the binding of MAb BW 494 to MAb BW 705.

Attempts are underway, using this internal image anti-paratope MAb BW 705 (Ab2B), to generate a tumour specific vaccine for the adjuvant treatment of mucin expressing human carcinomas.

### Xenosensitisation induced by *in vivo* injection of murine monoclonal antibodies

Lucienne Chatenoud, J.C. Saccavini & J.F. Bach

*INSERM U25, Hôpital Necker, Paris, France and ORIS, Saclay, France.*

Murine monoclonal antibodies (MAb) specific for various cell membrane antigens are now widely used in clinical practice as immunosuppressive agents. They are also administered in clinical oncology for therapy and diagnosis. One major side effect of *in vivo* MAb injection is the immunisation that almost invariably occurs when MAbs are injected alone (i.e. in the absence of associated conventional immunosuppression). Since 1982 we have studied 75 renal allograft recipients treated for 14–30 consecutive days with the OKT3 MAb. Human anti-OKT3 antibodies produced (both IgG and IgM) exhibit a particular specificity since they exclusively recognise isotopic and idiotypic OKT3 determinants. Moreover this response was shown to be oligoclonal. Only the IgG anti-idiotypic component neutralises the MAb therapeutic effectiveness. These results were extended to other anti-T-cell MAbs and a similar response, restricted in terms of specificity and clonality, was found when analysing 23 rhesus monkeys treated with different anti-CD4 and anti-CD8 MAbs. More recently 40 patients with ovarian or colonic tumours that received only one or two 1 mg injections of labelled $F(ab')_2$ MAb fragments for immunoscintigraphy were analysed. Fifteen of them showed significant antibody titres 10–20 days following even only one MAb injection. These antibodies were anti-idiotypic thus inhibiting the binding of injected $F(ab')_2$ MAbs to their targets.

### Localisation of monoclonal antibody AUA1 and its $F(ab')_2$ fragments in human tumour xenografts: an autoradiographic and immunohistochemical study

S. Pervez, A.A. Epenetos, W.J. Mooi, D.J. Evans, G. Rowlinson, B. Dhokia & T. Krausz

*Department of Histopathology and ICRF Group, Hammersmith Hospital, London, UK.*

Monoclonal antibody AUA1 reacts *in vitro* by staining all tumour cells mainly on their surface membrane. To investigate the accessibility of these sites by antibody when the tumour is present as a solid mass *in vivo*, subcutaneous xenografts were prepared in nude mice. Mice were injected i.v. with $^{125}$I-labelled AUA1, $^{125}$I AUA1 $F(ab')_2$ or $^{125}$I-labelled HMFG2 (a negative control). Animals were killed at days 1, 3 and 6. Gross and microautoradiography and immunohistochemistry were performed on frozen and paraffin embedded tissues of tumour and normal organs.

The *in vivo* injected antibody, in contrast to the *in vitro* results, was localised only, on a thin layer of tumour cells positioned adjacent to vascularised stroma. On small microscopic sized tumour islands the antibody penetration was complete. Strikingly, most of the radioactivity was mainly on the cell membrane similar to that seen *in vitro*. With intact antibody, similar autoradiographic results were obtained at days 1, 3 and 6. With $F(ab')_2$ there was deeper penetration into the tumour at days 1 and 3, but by day 6 the activity had greatly decreased. Radioactivity in the control organs was limited to the blood pool with no specific localisation. Negative control antibody HMFG2 showed no localisation in the tumour.

These results were not due to differences in antigenic expression by the tumour cells, as uniform staining was observed when AUA1 was applied to frozen or paraffin sections, but more likely reflect the problem of accessibility of antigenic sites *in vivo*.

### The microdosimetry of alpha emitters in radioimmunotherapy

D.R. Fisher

*Health Physics Department, Pacific Northwest Laboratory, PO Box 999, Richland, Washington 99352, USA.*

Radiation dosimetry is important both for treatment planning and for evaluating the success of radiation therapy. Biological response to alpha radiation may be highly variable at a given absorbed dose, depending on the local distribution of alpha-particle energy. Predicting the effectiveness of alpha-particle radiation in radioimmunotherapy depends on adequately characterising the distribution of energy at the cellular level. Four factors characterise the microdosimetry of alpha-particle interactions with living matter: (1) the average (absorbed) dose; (2) the probability density in specific energy (or distribution of doses to small targets); (3) the delta function (or fraction of targets completely missed); and (4) the mean number of hits per target. Determination of these elements requires careful consideration of the spatial distribution of alpha-emitting sources with respect to targets, the initial alpha-particle energy, the number of emissions per source, the relative specific ionisation as a function of track length and the size of the target (such as the diameter of the cell nucleus). Although precise correlations have not yet been developed between probability density in specific energy and the tumour-eradication effectiveness of alpha-emitting radioimmunoconjugates, such relationships will become apparent with further experimentation and the careful application of microdosimetric principles.

### Blood clearance of immunotoxins made with different toxin A-chain and ribosome-inactivating proteins

E.J. Wawrzynczak, A.J. Cumber, R.R. Henry, J. May, G.D. Parnell, N.R. Worrel & J.A. Forrester

*Institute of Cancer Research, Sutton, Surrey, UK.*

Immunotoxins were synthesised by attaching ricin A-chain, abrin A-chain, and two ribosome-inactivating proteins (RIPs), gelonin and momordin, to a single monoclonal antibody LICR-LOND-Fib 75. All four immunotoxins were powerfully and specifically cytotoxic to the human EJ bladder carcinoma cell line: protein synthesis by EJ cells growing in tissue culture was inhibited by 50% at immunotoxin concentrations ranging between $7\times10^{-11}$M and $5\times10^{-10}$M.

The blood clearance of immunotoxins after intravenous administration to normal rats was measured in the serum using a solid-phase ELISA specific for each type of toxin A-chain or RIP. A substantial proportion of Fib 75-ricin A was lost from the circulation within 2 min of injection consistent with hepatic recognition of oligosaccharide chains present on ricin A-chain. In contrast, the immunotoxin containing abrin A-chain, which is not glycosylated, was cleared much less rapidly so that the serum concentration of Fib 75-abrin A was 7.5-fold higher than that of Fib 74-ricin A after 24 h. Fib 75-gelonin and Fib 75-mormordin persisted in the bloodstream at levels intermediate between those of the A-chain immunotoxins. However, the B-phase half-life on clearance of the two RIP immunotoxins was similar to that of Fib 75-ricin A ($t_{1/2}$, 8 h), contrasting with a longer half-life for Fib 75-abrin A ($t_{1/2}$, 15 h). This suggests that oligosaccharide residues present on gelonin and momordin may contribute to the clearance of immunotoxins containing these RIPs.

### Tumour site activation of cytotoxic agent

K.D. Bagshawe, F. Searle, C. Springer, J. Boden, R. Melton, R. Sherwood & M. Jarman

*Cancer Research Campaign Laboratories, Department of Medical Oncology, Charing Cross Hospital, London W6 8RF, UK.*

The distribution kinetics of antibodies and antibody-toxin conjugates result in 'areas under the curve' which are less favourable for the delivery of toxic substances to tumours than is required for major therapeutic advance. The specificity of antibodies can be used more effectively by delaying the delivery of a toxin to a time when the tumour/non-tumour concentration ratios are most favourable and this requires 'capture' of the toxin or 'activation' of the toxin at the tumour site. It would also be advantageous if some form of amplification could be achieved at tumour sites.

These considerations have led to a two-phase approach. In the first phase a conjugate comprising an antibody fragment coupled to a non-mammalian enzyme is injected and allowed to localise at tumour sites. This is followed after an appropriate interval by a prodrug characterised by being readily diffusible at least through extracellular fluids and by being relatively stable by virtue of a protective group which acts as substrate for the targeted enzyme. Enzymatic action converts the product into a highly toxic agent which can diffuse readily through tumour extracellular fluids and enter cells but which ideally has a short half-life so that it reaches normal renewal tissues only in limited amounts.

Experimental data confirm the feasibility of this approach.

### Immunotherapy with monoclonal antibodies

G. Schulz, M. Buchler, K.H. Muhrer, R. Klapdor, D.W. Beelen & K. Bosslet

*Research Department, Behringwerke, Marburg, Department of Surgery, University of Ulm and Giessen, Department of Internal Medicine, University of Hamburg and Essen, FRG.*

MoAbs may induce destruction of tumour or other target cells by ADCC, CMC or phagocytosis of opsonised circulating cells. Clinical data demonstrate that these mechanisms operate in patients. In a phase I/II trial 10 patients with GvHD grade III after allogeneic BMT have been treated with a murine MoAb of IgG2b (BI 51.0113) isotype directed against the human T-cell receptor. Seven out of 8 evaluable patients showed improvement of symptoms after a 5-day therapy. In another phase I/II trial, 35 patients with advanced pancreatic cancer were treated with MoAb 494/32 (BI 51.011) which mediates ADCC and inhibits functional properties of human pancreatic cancer cells. Total doses up to 720 mg were well tolerated when applied within less than 14 days. Out of 30 evaluable patients, 2 (6.6%) showed a mixed response, 8 patients (26.7%) had stable disease up to 39 weeks (median = 24 weeks), and 20 patients (66.7%) had progressive disease. Eighteen out of 23 patients developed HAMA. Analysis of the human IgG anti-MoAb 494/32

response demonstrated that 3 out of 4 patients produced anti-idiotypic IgG. Preincubation of effector cells with rh GM-CSF enhanced ADCC activity *in vitro*. Patients treated with rh GM-CSF in a phase I/II trial demonstrated as rise in mature neutrophils, eosinophils and monocytes above pre-treatment levels. Thus a combination of MoAb 494/32 and GM-CSF result in better tumour response in patients with pancreatic cancer.

**Conversion of xenogeneic monoclonal antibodies to specific tolerogens**

A.H. Sehon

*MRC Group for Allergy Research, Department of Immunology, The University of Manitoba, Winnipeg, MB, Canada, R3E OW3.*

The therapeutic effectiveness of xenogeneic monoclonal antibodies (XIg) or of their conjugates with toxins (XIg-Tx) is undermined by their inherent immunogenicity. The coupling of an optimal number of monomethoxypolyethylene glycol (mPEG) chains (M.wt 3,000–6,000) on to XIg results in tolerogenic derivatives. The test model in this study consisted of different inbred strains of mice which received injections of tolerogenic mPEG conjugates of human monoclonal (myeloma) immunoglobulins, HIgG(mPEG), and immunogeneic doses of heat-aggregated HIgG(ha-HIgG). Administration of mPEG conjugates of HIgG seven days prior to immunization with ha-HIgG resulted in a marked suppression (~90%) of murine IgG, IgM and IgA antibodies to HIgG. The suppression could be maintained for periods in excess of 250 days by multiple, alternating injections of (HIgG)mPEG and Ha-HIgG. The suppression was dose-dependent and the state of tolerance could be transferred with spleen cells of immunosuppressed mice to syngeneic recipients, as well as by freeze/thaw extracts of these cells. The possible mechanism underlying the immunosuppression by mPEG conjugates may involve the activation of suppressor T-cells. The conjugates of XIg are expected to be devoid of antigen binding capacity because of conformational changes and steric hindrance due to the coupled mPEG chains. Hence, for therapeutic purposes it is envisaged that it will be necessary to administer tolerogenic mPEG conjugates of XIg or XIg-Tx prior to treatment with the corresponding non-pegylated XIg or XIg-Tx.

**Dosimetry of radiolabelled antibodies**

T.E. Wheldon

*Beatson Oncology Centre, Belvidere Hospital, Glasgow G31 4PG, UK.*

Cancer treatment using radiolabelled antibodies is a new form of therapy. As with conventional radiotherapy, success will depend on the dose received by critical normal tissues. Compartmental models may be used to calculate radiation dose to tumour and various organs. Tumour volume is an important parameter whose accurate estimation is difficult. This can be by-passed if we know the uptake of radionuclide in tumours excised after tracer administration. Estimation of dose to micrometastases requires the use of mathematical models which take account of energy dissipation outwith the tumour. Microdosimetric models are less helpful in assessing dose to normal tissues. For systemic administration of radiolabelled antibodies, bone marrow is the dose-limiting organ. However, regional treatment strategies are developing for which non-haemopoietic organs may be critical. Also, bone marrow might be used to obviate haemopoietic damage. It is likely that doses to non-haemopoietic organs such as liver will assume increasing importance. It is important that physical dose calculations be supplemented by radiobiological models which incorporate dose rate effects. This is needed to relate the 'tolerance dose' of an organ to clinical experience using external beam fractionated radiotherapy. Information obtained from pre-therapeutic trace studies, should enable prediction of achievable tumour and normal tissue doses, and the prospective selection of patients who may benefit from antibody-targeted radiotherapy.

**Dose fractionated radioimmunotherapy of lymphoma with $^{131}$I Lym-1**

Sally J. De Nardo, G.L. De Nardo, D.J. Macey, S.L. Mills, L.F. O'Grady, J.P. McGahan, E. Hu & A.L. Epstein

*University of California Davis Medical Center, Sacramento, USA.*

Multidose $^{131}$I Lym-1, and IgG2a MoAb produced against Burkitt's lymphoma has been used to treat 16 patients with aggressive B-cell lymphoma and 2 patients with lymphocytic leukaemia. Ninety-five doses of $^{131}$I Lym-1 have been given (1–9 per patient) at 2–6 weeks intervals up to 330 mCi. Each treatment (Rx) consisted of 5–50 mg unlabelled Lym-1 preceding 30–60 mCi of $^{131}$I Lym-1 (3–6 mg). Response was classified as: CR = no detectable disease; PR = tumour regression >70%; SR = tumour regression >30% <70%; NR = tumour regression 0–30%.

The patients with leukaemia and nodal disease had PR after 5 and 2 treatments. Of 16 patients with solid disease, 2 had CR, 5 had PR, and 4 had SR. Of the 5 non-responders, 3 died after one Rx and one developed HAMA after 2 Rx. Three of 18 patients have developed HAMA. Most patients have had no toxicity; transient fever, myalgia or hives occurred in 6 patients, hypotension in one, and a moderate decrease in platelets occurred in 3 patients. Radiation doses calculated per mCi ranged from: tumour 2–50, whole body 0.3–0.5 and marrow 0.5–1.2 rads. We conclude that radiolabelled monoclonal antibodies can be used to treat patients with B-cell malignancies without significant toxicity, and in striking contrast to the toxicity associated with chemotherapy.

The marked response rate in the face of only modest toxicity in this study encourages the development of subsequent trials.

**Intraperitoneal (i.p.) radioimmunotherapy with $^{131}$I- and $^{90}$Y-labelled monoclonal antibodies**

J.S.W. Stewart, V. Hird and A.A. Epenetos

*ICRF Group, Hammersmith Hospital, London, UK.*

Pharmacokinetics of i.p. radiolabelled antibodies have been studied in 35 ovarian cancer patients. Eleven received $^{131}$I and 19 patients $^{90}$Y-labelled antibody (5 also receiving i.v. EDTA). Five patients received $^{131}$I-labelled antibody six weeks after $^{90}$Y-labelled antibodies. These patients had developed antibodies to mouse immunoglobulin (HAMA). Activity ranged from 100 to 157 mCi $^{131}$I and 5 to 20 mCi $^{90}$Y. Patients had measurements of blood and urine activity. Lithium fluoride thermoluminescent dosimetry (TLD), was used to estimate the non-specific radiation to the peritoneum in 21 patients. Bone marrow dose was estimated using the MIRD formulation.

In HAMA negative patients, marrow toxicity was the dose limiting toxicity. Peak blood activity was seen at 40 h with 26% and 21% of $^{131}$I- and $^{90}$Y-labelled antibody in the circulation. More than 65% of $^{131}$I but less than 12% of

$^{90}Y$ was excreted in the urine. Marrow suppression in patients receiving $^{131}I$ was predicted by our model but was not predictable in patients receiving $^{90}Y$-labelled antibodies. Additional marrow irradiation from $^{90}Y$ in bone may account for this.

HAMA positive patients had less than 5% of the injected activity in the blood and rapid urinary excretion of $^{131}I$. The urine excretion of $^{90}Y$ was increased three-fold by intravenous EDTA. The radiation dose to the peritoneum was less than 600 cGy, and is unlikely to contribute to tumour response or local toxicity.

Marrow irradiation is the dose limiting factor with both $^{131}I$- and $^{90}Y$-labelled antibodies. The dissimilar pharmacokinetics and dosimetry of the isotopes demand different solution to decrease toxicity and increase efficacy.

**Biodistribution of $^{131}I$-labelled monoclonal antibodies in human colon tumours by an *ex vivo* perfusion model**

E. Kraas, E. Lohde, O. Abri, H. Schlicker, S. Matzku, H. Kalthoff, W. Schmiegel & I. Chirl

*Abtl. u Isotop. Abtl. Berlin, DKFZ Heidelberg, Klin. Immunologie, Hamburg, West Germany.*

The use of monoclonal antibodies for diagnostic and therapeutic purposes depends on the uptake and retention of the antibodies in the target neoplastic tissues. The purpose of the study was to examine the biodistribution of specific and non-specific antibodies in resected human colon tumours.

After resection of the colon tumour, the supplying artery was canulated and perfused with fresh frozen plasma and Heparin. Continuous control of pressure, flow, temperature, pH and various metabolic parameters were performed after administration of $^{131}I$-labelled anti-CEA antibody. Highly differentiated adenocarcinomas of the colon showed a much higher antibody uptake, than undifferentiated tumours. Between 3 and 7% of the injected antibody was found in the tumour tissue. Autoradiography showed inhomogeneous binding in the tumour tissue. The non-specific antibody-perfusion showed no tumour binding.

We conclude that the *ex vivo* perfusion of resected colon carcinomas can be used to measure the kinetics of binding and clearance of monoclonal antibodies in tumour tissue by direct scintigraphy. The cellular biodistribution of the antibody can be documented through autoradiography.

**Monoclonal antibodies against individual cytokeratins in the detection of metastatic spread**

J. Kovarik, A. Rejthar, L. Lauerova & B. Vojtesek

*Department of Immunology, Inst. Medical Research, Brno, Czechoslovakia.*

A panel of 17 monoclonal antibodies recognising epitopes of keratins 7, 8, 18 and 19 was screened on bone marrow aspirates and lymph node biopsies of patients with lymphoproliferative malignancies and non-malignant diseases to define their reactivity with a non-epithelial structure. The results revealed that only a limited number of antibodies exerted constantly no staining of bone marrow elements. Three antibodies showed a certain degree of positivity confined exclusively to bone marrow cells of patients with lymphoproliferative neoplasms, while another 6 antibodies exhibited non-specific binding in some smears regardless of their origin. With all antibodies there were positive reactions with lymph nodes. Staining restricted to keratins 8 and 18 was noted in the extra-follicular reticular cells of all nodes. Surprisingly, a non-epithelial positive granular staining could be found with all antibodies in the histiocytic reticular cells but only in uninvolved lymph nodes of non-Hodgkin's lymphoma patients. Morphological complementation and selection of antibodies is necessary in order to avoid false results when such reagents are used to detect carcinoma metastatic cells.

**A phase I study using iodine-131 labelled OC 125 in the treatment of epithelial ovarian carcinoma**

N.J. Finkler, M.G. Muto, G. Muto, A. Kassis, C.T. Griffiths, S. Tumeh, V.R. Zurawski Jr. & R.C. Knapp

*Brigham and Women's Hospital, 75 Francis Street, Boston, MA, USA.*

The monoclonal antibody (MAb) OC 125 has been shown to react with approximately 80% of ovarian cancers. Preliminary investigation using iodine-131 ($^{131}I$) labelled OC 125 (1–2 mCi) given intraperitoneally or intravenously to patients with either primary or recurrent ovarian cancer indicates that intraperitoneal administration of antibody conjugate results in higher concentration of recovered antibody in tumour.

A phase I clinical trial was carried out utilising $^{131}I$-labelled OC 125 given intraperitoneally to patients with recurrent epithelial ovarian carcinoma who have failed conventional therapies. To date, 8 patients have been infused with the radiolabelled antibody. Each patients received a single injection of an escalating $^{131}I$-MAb dose.

All treatments have been well tolerated and without clinically apparent side effects. No haematopoietic or hepatic toxicity has been observed. All eight patients are alive. Two patients evaluable for response have had documented decreases in tumour burden; one patient had a 50% reduction in tumour along the small bowel mesentery.

We conclude that $^{131}I$-labelled OC 125 may be safely administered intraperitoneally to patients with recurrent ovarian cancer and that toxic doses have not yet been met.

**Therapeutical application of radiolabelled monoclonal antibodies: preliminary experiences in 25 patients with solid tumours**

P. Riva & S. Lazzari

*Nuclear Medicine Department and Inst. Oncologico Romagnolo Health Phisic Department, M. Bufalini Hospital, Cesena, Italy.*

Twenty-five patients with advanced neoplastic disease (3 lung, 2 breast, 10 ovarian and 10 colon cancer) were submitted to radioimmunotherapy following the failure of chemotherapy and radioimmunotherapy. The antibodies employed were HMFG1, HMFG2, AUA1, H17E2 (ICRF, London), BW494/32 (Beheringwerke, FRG), F023C5 and B72.3 (Sorin Biomedica, Italy). These were chosen on the basis of immunostaining and immunoscan results and were labelled with $^{131}I$ (mean dose 92 mCi). The MoAbs were administered intravenously (4 patients), intrapleurally (1 patient) and intraperitoneally (20 patients). Thirteen patients underwent two or more treatments. No early or late adverse effects were encountered. All patients developed antimouse antibodies (HAMA) which reduced, during subsequent courses in the efficacy of the therapy. The calculated dose delivered to the tumours ranged from 2,000 to 6,300 cGy (mean 3,440 cGy). We observed no responses in 9 patients and partial responses in 4 patients. In 5 patients there was stabilisation of disease. Significant clinical improvement and evidence of tumour volume reduction (30–50%) persisting from 3 to 11 months was achieved in 5 cases. Finally a complete response lasting from 11 to 15 months was obtained in 2 patients.

**Treatment of patients with B-cells malignancies with anti-CD19 monoclonal antibodies**

Anke Honselaar, A. Hekman, J. Sein, Th. Vroom, S. Israels, R. Somers, W. ten Bokkel, Ph. Rumke & C. Meief

*The Netherlands Cancer Institute, Amsterdam and Slotervaart Hospital, Amsterdam, The Netherlands.*

Five patients with B-cell non-Hodgkin's lymphomas were treated with the mouse monoclonal antibody CLB-CD19, subclass IgG2a, directed against the B-cell specific antigen CD19, not expressed on bone marrow stem cells or plasma cells. The purpose of this phase I/II clinical trial was to determine pharmacokinetics and toxicity. Four patients were treated with 225 mg MoAb intravenously in an escalating schedule over four consecutive days. One patient received 60 mg MoAb twice weekly during a period of 3 weeks. Treatment was well tolerated. Peak levels of plasma MoAb ranged from 30 to 70 $\mu g ml^{-1}$. The plasma half-life of the MoAb was 22.5 to 60 h. The MoAbs could be demonstrated in the plasma until 10 days after end of treatment. After each infusion there was a transient decrease in the number of B-lymphocytes. Remaining B-cells were partially modulated. Homing of the antibody was found in the bone marrow, lymph nodes and lymphoma of the skin. Peripheral lymphocytes were saturated with MoAbs. Complete saturation was reached in the bone marrow whilst the cells in lymph nodes were only partially coated with MoAbs. None of the patients developed antibodies against the mouse immunoglobulin. No short or long-term effects were seen in serum immunoglobulin levels. In one patient a reduction of 43% in peripheral B-lymphocytes was maintained during 6 weeks after treatment. Clinically two patients showed a minimal tumour regression during a period of 3 months.

**Isotype variants of CAMPATH-1 antibody: requirements for effective antibody action *in vivo***

M.J.S. Dyer, G. Hale, F.G.J. Hayhoe & H. Waldmann

*Departments of Haematological Medicine and Immunology, University of Cambridge, UK.*

A series of rat monoclonal antibodies against a non-modulating antigen (CAMPATH-1) has been produced. All are lytic with human complement but only the IgG2b antibody (CAMPATH-1G) is able to elicit antibody-dependent cellular cytotoxicity with human effector cells. Twenty patients have received CAMPATH-1G. Normal and malignant lymphocytes were cleared from the peripheral blood in all cases. Bone marrow infiltration was cleared in 5 out of 12 assessable cases. Splenomegaly was reduced or resolved in all 5 cases. Less sensitive were lymph nodes. CAMPATH-1G is not effective intrathecally. The combination of non-modulating antigen and correct antibody isotype allow more effective immune antibody action in man.

**Radioimmunolocalisation using a mouse monoclonal antibody reacting with rat breast and colonic cancer**

Louise Baker, C.Y. Yiu, M.J. O'Hare & C.G. Clark

*Department of Surgery, University College, London and Institute of Cancer Research Sutton, Surrey, UK.*

In the study of immunotoxin effect on tumour, a syngeneic animal tumour model is preferable to a xenograft model as the former should provide a better reflection of immunotoxin behaviour when used in the clinical context. However, few syngeneic models have so far been used for this purpose. An IgG1 mouse monoclonal antibody (JB28.4) has been raised against a rat colonic cell line RCC2 which was derived from a transplantable Fischer rat colonic cancer 4047. The antibody shows a restricted normal tissue distribution in the rat. It reacts with the cell surface of the transplant and also with the cell surface of a rat breast cancer cell line MAT B3. Both tumours can be grown subcutaneously in Fischer rat. A radiolocalisation study of JB28.4 to these tumours was performed using $^{125}I$-labelled antibody. 1.5–2 cm tumour bearing rats were injected intravenously with 8–10 MBq (12 $\mu g$) of labelled antibody and sacrificed at 24, 48 and 72 h. An isotype matches mouse monoclonal LICR-LON M8 which was non-reactive with the tumours was used as a control. A significant uptake of antibody as measured by the tumour blood ratios was observed at all time points with the breast cancer as compared with the control ($P<0.001$), but only at 48 h ($P<0.01$) for the colonic tumour.

**Expression of human cell surface antigen defined by AuA1 monoclonal antibody in colon cancer and polyps**

A. Bamias, D.V. Skarlos, Penelope Paizi-Biza, E. Samadas, G. Aravantinos, Ekaterini Anagnostaki & C. Kittas

*Agii Anargyri Cancer Hospital and Department of Pathology, Athens University, Greece.*

AuA1 monoclonal antibody (ICRF) was derived by immunising BALB/c mice with the colon adenocarcinoma cell line LoVo. It recognises an epithelial, cell surface protein, coded by MIC18 gene on chromosome 2. AuA1 reacts with a wide range of epithelial tumours and normal tissues.

In this study, 15 colon cancers of different grading and 15 polyps were stained. Paraffin embedded sections 5 $\mu m$ were stained with Haematoxylin and Eosin, and immunohistochemically, with AuA1 using the indirect immunoperoxidase method. A synchronous staining of normal colon tissue, polyps and cancer was performed.

Seventy-eight to 90% of cancer cells showed a very strong reaction with AuA1 monoclonal antibody. This did not correlate with the grade of the tumour. Polyps showed weaker reaction with less than 30% of cells being positive. Micrometastases, not detected by conventional methods, were revealed by immunohistochemical technique in certain cases.

**Biodistribution of indium-111/A5B7 conjugates in a xenograft model**

P. Barnett, R.G. Buckley, P.J. Burke & F. Searle

*Cancer Research Campaign Laboratories, Department of Medical Oncology, Charing Cross Hospital, London W6 8RF, UK.*

An anti-CEA antibody A5B7 has been conjugated with DTPA (using *N*-hydroxysuccinamide) and DHDE (*N,N'*-di(hydroxycarbonylmethyl)-*N,N'*((2-hydroxy)hydroxycarbonylbenzyl)diaminoethane) (using a water soluble carbodiimide).

Radioimmunoassay and FPLC studies showed that the conjugation procedure has not impaired the CEA binding ability of the antibody or induced aggregate formation. Biodistribution and imaging studies of indium-111 labelled conjugates have been carried out in nude mice bearing the human colon carcinoma xenograft LS-174T.

Results showed favourable tumour to tissue ratios for the DTPA conjugate, enabling tumours to be visualised by gamma-camera imaging. Tumour to tissue ratios for the DHDE conjugate were less favourable.

### Induction of expression of glia- and glioma-associated antigens (GAA) in human glioma cell lines

T. Bilzer, D. Stavrou & W. Wechsler

*Department of Neuropathology, University of Dusseldorf and Institute of Pathology, Hospital München-Bogenhausen, FRG*

Heterogeneity at the cellular antigen level is the basis of major problems in neuro-oncology, especially in immunodiagnosis and therapy of malignant human gliomas (WHO grades III and IV). As identified by monoclonal antibodies (McAbs), anaplastic gliomas are composed of various cell subpopulations which may change their antigenicity during progressive growth. Permanent cell lines and clones of such gliomas have the tendency to develop into cell populations which only partly represent the antigenicity of the primary tumour.

In uncloned cell lines of two glioblastomas (87HG-28, 87HG-39) and an astrocytoma grade III (86HG-31), respectively, the amount of GAA defined by two McAbs (Muc 2-39, Muc 2-63) against epitopes on 75 and 96kD glycoproteins of the cell surface membrane decreased with increasing number of *in vitro* passages. In this cells, immunocytochemistry and Western blots revealed the presence of GAA within the cytosol. Furthermore, cells were characterised by an accelerated proliferation rate, an increased saturation density and a loss of differentiation markers such as the glial intermediate filament protein GFAP. After treatment with db-cAMP, GAA-properties, expression of differentiation markers as well as morphological and proliferation parameters were widely rearranged, as illustrated by the reappearance of GAA on the cell surface, expression of GFAP and extension of cell processes.

Our results suggest approaches to modulate the presence and responsiveness of antigenic determinants in poorly differentiated cells of anaplastic human gliomas by the application of chemicals acting as, or by influence on, their second messenger systems.

### A comparison of antibodies to carcinoembryonic antigen (CEA) and epithelial membrane antigen (EMA) in human colorectal cancer

B.R. Davidson, C.Y. Yiu, J. Styles, M. Ormerod, C.G. Clark & C. Dean

*Department of Surgery, University College, London and Institute of Cancer Research, Sutton, UK.*

We compared two high affinity MAbs, one to CEA(C46) and one to EMA(ICR2) in tissues obtained from 31 patients with cancer, 14 patients with polyps and 17 with normal colon. Immunohistochemistry was used and the results classified as positive or negative. Staining was heterogeneous for both antibodies.

| | *Staining reaction* | |
|---|---|---|
| | *EMA(ICR2)* +*ve* (%) | *CEA(C46)* +*ve* (%) |
| Carcinoma ($n=18$) | 15 (83) | 18 (100) |
| Nodal metastases ($n=6$) | 5 (83) | 5 (83) |
| Liver metastases ($n=7$) | 5 (71) | 4 (57) |
| Polyps ($n=14$) | 1 (7) | 11 (79) |
| Normal colon ($n=17$) | 2 (12) | 12 (71) |

The anti-EMA antibody reacts with slightly fewer colonic cancers than the anti-CEA antibody (83% *vs.* 100%). The anti-EMA antibody has fewer reactions, however, with normal colon (12% *vs.* 71%) and benign polyps (7% *vs.* 79%) than the anti-CEA.

### Diagnostic and clinical value of immunoscintigraphy with the F(ab′)$_2$ fragment of the monoclonal antibody 225, 28S in malignant melanoma

Heidi Dazzi, Susan Owens, T. Cerny, Shirley Mackenzie, Pamela Nuttall & N. Thatcher

*Department of Medical Oncology and Reg. Department of Medical Physics and Bioengineering, Christie Hospital and Holt Radium Institute, Manchester, UK.*

The monoclonal antibody 225, 28S to the high molecular weight melanoma associated antigen has been tested in patients with various stages of malignant melanoma. A total of 70 immunoscintigrams have been performed in 63 patients, using the Tecnemab-1 kit (Sorin Biomedica). In these patients 73 of 113 previously known lesions were observed (64.4%) and in addition 18 lesions which were previously unknown were detected. These lesions were later confirmed by further investigations.

Detailed analysis of the different sites of the metastases shows that immunoscintigraphy is helpful for detecting tumour in deep lymph nodes and lower intra-abdominal masses. In skin and subcutaneous tissue, only larger lesions were detected (>2cm). Diagnostic information obtained for pulmonary and gastrointestinal disease is very poor. Non-specific accumulation was present in the liver, spleen, kidney, cardiac area, bone marrow, colon and scrotum, as well as in recent surgical scars and degenerative bone lesions.

We conclude that this procedure is not indicated for routine screening for metastatic disease in patients with malignant melanoma. For special problems, such as the involvement of deep lymph nodes or ocular melanoma, this technique may provide important information and could, therefore, be indicated as an additional investigation.

### Isolation and biochemical characterisation of a $M_r$ 48,000 glycoprotein recognised by BCD-E8 and BCD-F2 monoclonal antibodies

Brigitte Grouix, A. Amarouch, R. Jolicoeur & Rosemonde Mandeville

*Immun. Res. Center, Institut Armand-Frappier, Laval-des-Rapides, Quebec, Canada H7N 4Z3.*

Murine monoclonal antibodies BCD-E8 and BCD-F2 were prepared against the human breast carcinoma cell line BT-20. A soluble antigen recognised by these antibodies was obtained by mechanical disruption of the MCF-7 carcinoma cells. By immunoprecipitation and immunoblotting, we were able to isolate the antigen recognised by BCD-E8 and BCD-F2. This antigen was sensitive to periodate oxidation and B-glycosidase digestion, demonstrating the carbohydrate nature of the epitope. Moreover, lectin-absorption experiments indicated that BCD-E8 and BCD-F2 recognise oligosaccharide sequences containing *N*-acetyl-D-glycosamine residues. We conclude that both BCD-E8 and BCD-F2 recognise a glycoprotein with the same molecular weight and the same side chain.

### Increased CA125 elevation in patients after administration of $^{131}$I-OC125 caused by human anti-mouse antibodies

H.J. Haisma, K.R. Moseley, R.C. Knapp, E. Boven & H.M. Pinedo

*Departments of Oncology, Dana Farber Cancer Institute, Boston, USA and Free University Hospital, Amsterdam, the Netherlands.*

CA125 is a valuable tool for monitoring disease in patients with ovarian cancer. Recently, imaging and therapy studies have been initiated using antibody OC125. We injected $^{131}$I-labelled OC125 $F(ab')_2$ i.v. or i.p. to localise the tumour lesions in patients. On analysis of CA125 levels from 17 patients before and at least 1 month after injection of antibody, 9 patients (54%) had values that had increased to >5,000 CA125 U $ml^{-1}$, which could not be explained by progressive disease. Such high levels were previously observed in 19 out of 4,500 (0.8%) samples from untreated patients. In the patients who were injected, CA125 levels started to rise 4 weeks after injection and remained elevated for 3 to 24 months. Nine patients had an anti-OC125 antibody response and 7 of them had CA125 levels above 5,000 U $ml^{-1}$. Tumour localisation was not impaired by the antibody response. No correlation could be found between antibody response, antibody dose, route of administration and CA125 levels. After preincubation of the sera with protein A-Sepharose to remove immunoglobulin, CA125 levels dropped precipitously in sera from patients with an antibody response, but not in sera from patients without an antibody response. We conclude that human anti-OC125 antibodies interfere with the CA125 assay. This phenomenon may also occur in patients where other monoclonal antibodies are used both for measuring circulating antigen and for *in vivo* studies. We demonstrated that in the presence of human anti-mouse antibodies, CA125 values can be corrected by removing immunoglobulin from the serum.

**Preparation of mouse monoclonal antibodies and their $F(ab')_2$ fragments for *in vivo* application**

Leena Hakalahti, Riitta Kurkela & Pirkko Vihko

*Biocentre and Department of Clinical Chemistry, University of Oulu, Finland.*

Mouse IgG1 monoclonal antibodies (MAbs) directed against human prostatic acid phosphatase were used for radioimaging of prostatic cancer metastases in our laboratory. We have developed two different two-step methods to purify these MAbs from cell culture media containing 1–5% fetal calf serum. Methods are based on ion exchange and hydrophobic interaction chromatographies. The main contaminants, bovine albumin and transferrin are effectively removed by these methods. Purity analyses made by SDS-PAGE and sterility and pyrogen tests show that these MAb meet the requirements given by FDA for MAbs used for *in vivo* purposes.

We have also digested IgG1 to $F(ab')_2$-fragments using pepsin and papain digestions. Papain digestion was shown to be more efficient in $F(ab)_2$-fragmentation for radioimaging purposes. The yield of $F(ab')_2$-fragments from papain digestion was $50 \pm 5\%$ of the theoretical maximum. Both pepsin and papain digestions gave $F(ab')_2$-fragments with immunoreactivity and affinity identical to those of the original IgG1.

**Monoclonal antibodies Ki-M3 and Ki-M7 react with renal cell carcinomas**

M.-L. Hansmann, U. Kaiser, I. Papadopoulus & H.J. Radzun

*Pathologisches Institut der Universität Kiel and Urologische Universitäts-Klinik, Kiel, FRG.*

The monoclonal antibodies Ki-M3 and Ki-M7, which detect macrophages and monocytic cells, were tested using immunohistochemistry on a series of benign and malignant human tumours ($n=50$). Positive reactivity was detected in renal cell carcinomas ($n=20$), a few cases of breast carcinoma and in one case of prostatic carcinoma. In normal human kidney, Ki-M3 and Ki-M7 reacted with proximal and parts of the distal tubules of the Henle limb. Monoclonal antibodies Ki-M3 and Ki-M7 proved useful in detecting renal cell carcinomas and in distinguishing these tumours and their metastases from morphologically similar neoplasm.

**Radio silver ($^{111}$Ag) separation for labelling monoclonal antibodies for radioimmunotherapy**

D.K. Hazra, V.L. Lahiri, M. Kumari, P. Khanna & B. Arvind

*SN Medical College, Agra, India.*

$^{111}$Ag by virtue of its 7.45 days $T_{1/2}$ and beta emission appeared to be a candidate radionuclide for radioimmunotherapy. We describe the preparation of radio silver from $^{110}$Pd and the separation of $^{111}$Ag from $^{111}$Pd by anion exchange chromatography.

$^{110}$Pd was irradiated for 24 h in a neutron flux of $1 \times 10^{12}$ n $cm^{-2}$ $s^{-1}$ at the APSARA reactor BARC, Bombay. Six hours after irradiation the sample was dissolved in 10 ml 10 M HCl and applied to a Dowex-1 column (8% cross-linked 100–200 dry mesh, Sigma). The silver is eluted from this column using 10 M HCl and Pd is retained in the column. The product is over 99.5% pure and appears suitable for further processing for radiolabelling. The technique was earlier standardised using inactive Pd and Ag.

**Immunoreactivity of anti-HeLa monoclonal antibody**

V.L. Lahiri, D.K. Hazra, B.R. Elhence, K. Singh, M. Kumari, S. Sarah, B. Arvind & S. Khandelwal

*SN Medical College, Agra, India.*

A monoclonal antibody, anti-HeLa, was developed against HeLa cell line (Human Ca Cervix line). The specific reactivity of the MoAb with epitope on the surface of the HeLa cells was confirmed both by ELISA as well as by indirect immunoperoxidase staining. Further quantitative reactivity with HeLa cells was confirmed by RIA. The reactivity of the anti-HeLa was tested against a panel of malignant tissues by indirect immunoperoxidase method. Anti-HeLa antibody showed very strong positivity with squamous cell carcinoma of cervix but showed only mild positive reaction with squamous cell carcinoma of buccal mucosa and oesophagus. Anti-HeLa antibody showed mild reactivity with non-malignant cervix. Anti-HeLa monoclonal showed reaction similar to that of anti-keratin antibodies reported by Debus (1982, 1984) and Ramackers (1983).

**Simultaneous dual energy (SDE) radioimmunoscintigraphy (RIS) in the study of colorectal cancer**

L. Jenkins, R. Iorns, M.A. Macleod & R. Leicester

*Nuclear Medicine Department, Royal Naval Hospital, Gosport, Hants PO12 2AA, UK.*

The aim of this pilot study was to examine the feasibility of using the SDE technique to enhance the information gathered from radioimmunoscintigraphic examination of patients with colorectal cancer. It was also hoped that the technique would lead to the detection of small tumour masses due to the increased sensitivity of the Tc-labelled nanocolloid. SDE planar and single photon emission computed tomographic (SPECT) images were obtained 4–6 days post infusion with Imacis-1 ($^{131}$I-labelled murine anti-CEA, anti-CA-19.9). The SDE method requires that an injection of $^{99}Tc^{m}$-nanocolloid is given 24 h prior to final imaging.

One patient had a negative scan and was clinically asymptomatic despite a rising serum CA-19.9 (125 U $ml^{-1}$). Three patients with rising levels of CEA and CA-19.9 had positive SDE scans. Malignancy was confirmed visually (endoscopy) and histologically. Two patients had primary tumours confirmed at operation. The third case was of particular interest in that Imacis-1 and nonocolloid accumulations were found at colonoscopy to correspond to polyps (3–5 mm). On external counting the iodine label was present in one metaplastic and one dysplastic polyp whilst the $^{99}Tc^{m}$-nanocolloid was present in all three polyps. These initial results are encouraging.

**Pre-targeted immunoscintigraphy in patients with non-small cell lung cancer (NSCC) using streptavidin conjugated HMFG1 and indium-111-labelled biotin**

H.P. Kalofonos, D.J. Hnatowich, M. Ruschowski, D. Snook, G.B. Sivolapenko, J.P. Lavender & A.A. Epenetos

*ICRF Oncology Group, Hammersmith Hospital, London, UK and University of Massachusetts, USA*

We describe a new method for *in vivo* labelling monoclonal antibodies using streptavidin conjugated monoclonal antibody given first (pre-targeted), followed by indium-111-labelled biotin. We studied four patients with lung cancer (NSCC) to identify toxicity, localising efficiency, biodistribution and pharmacokinetics. We administered i.v. unlabelled HMFG1 antibody conjugated with streptavidin followed 3 days later by indium-111-labelled biotin. Twenty-four hours after $^{111}$In-biotin administration the uptake by each kidney was 5.0% and by liver 2.0% of the injected dose. Maximum tumour concentrations were achieved in 2 h but the optimal time for imaging was at 24 h with higher tumour to normal lung ratios.

Radioactivity in the serum had a $T_{1/2a} = 2$ min and $T_{1/2b} = 4$ h. The cumulative urinary excretion of radioactivity over 24 h was ~78% of the injected dose, of which 70% was excreted in the first 6 h. No toxicity was observed. These preliminary results indicate an improvement in tumour to normal tissue ratios, with the exception of kidneys.

**Kinetics, quantitative analysis and immunolocalisation using radiolabelled HMFG1-monoclonal antibody in patients with breast cancer**

H.P. Kalofonos, J.M. Sackier, M.N. Hatzistilianou, C.B. Wood, J.H. Waxman, P.J. Lavender & A.A. Epenetos

*Hammersmith Hospital, London, UK.*

HMFG1, IgG and F(ab')$_2$ fragments, radiolabelled with indium-111, were used to study patients with breast cancer. Twenty-four patients with primary breast cancer underwent tumour resection and quantitation of the radioactivity in tumour versus normal tissue following administration of specific and non-specific antibodies. The mean tumour uptake of HMFG1-F(ab)$_2$ fragments at 24 h was $5.8 \times 10^{-3}$% of injected dose per gram of tissue (% ID $g^{-1}$). This was higher than the intact antibody $2.6 \times 10^{-3}$% ID $g^{-1}$ ($P<0.05$), but at 48 h there was no significant difference, $5.7 \times 10^{-3}$ and $7.4 \times 10^{-3}$% ID $g^{-1}$ respectively. The mean tumour uptake with the specific antibody was higher than the non-specific antibody of the same subclass, $2.3 \times 10^{-5}$% ID $g^{-1}$ ($P<0.05$). Lymph node metastases, interestingly, showed higher antibody uptake than the corresponding primary tumours $8.0 \times 10^{-3}$% ID $g^{-1}$ ($P<0.05$).

Fifteen patients, were imaged using $^{111}$In-F(ab')$_2$ HMFG1-F(ab')$_2$ fragments. We observed positive localisation in 3 out of 7 with primary lesions, in 4 out of 6 with bone metastases, in 2 out of 4 with skin infiltration, in 1 out of 2 with lymph node and in 1 out of 2 with liver metastases. The optimal time for imaging was 48 h. All patients had significant concentration of $^{111}$In in the liver.

We conclude that HMFG1-F(ab')$_2$ fragments can localise specifically and faster than the intact IgG in breast cancer. However, this method needs further improvement prior to becoming a clinically useful method for staging breast cancer.

**Radiolabelled monoclonal antibodies for localisation and therapy of brain gliomas**

H. Kalofonos, T.R.B. Pawlikowska, D.E. Snook, P.J. Lavender, C.G. McKenzie, D.G. Thomas & A.A.Epenetos

*Hammersmith Hospital and Institute of Neurology, London, UK.*

Immunoscintigraphy was performed in 21 patients using iodine-123-labelled monoclonal antibodies against epidermal growth factor receptor (EGFR1) and placental alkaline phosphatase (H17E2). Ten patients were also imaged using a non-specific antibody (11.4.1) of the same immunoglobulin subclass. The specificity of targeting was confirmed by comparing imaging obtained by specific and non-specific antibodies. In addition, quantitative evaluation of the antibody uptake in tumour versus normal tissue was performed in 7 patients using the paired antibody technique, following tumour resection.

Seven patients with recurrent brain glioma (grade III or IV) who previously showed good localisation of radiolabelled antibody were treated with 40–140 mCi of iodine-131-labelled antibody delivered to the tumour area intravenously or by infusion into the internal carotid artery. Four patients showed clinical improvement lasting from 6 months to 2 years. One patient continues in remission 2 years after therapy but the other 3 who responded initially, relapsed 6–12 months after therapy and died.

No major toxicity was attributable to antibody guided irradiation. The patient who received 140 mCi developed a moderate but reversible thrombocytopenia and neutropenia. These promising but preliminary results should be explored further in randomised trials.

**Enhancement of monoclonal antibody binding after irradiation of tumour in a xenograft model**

H.P. Kalofonos, G. Rowlinson, A.W.J. Stuttle & A.A. Epenetos

*Imperial Cancer Research Fund Oncology Group, Hammersmith Hospital, London, UK.*

Nude mice bearing subcutaneous human colorectal carcinoma (LoVo), were exposed to single doses of external irradiation of 40 to 160 Gy, followed 24 h later by $^{111}$In-labelled monoclonal antibody. Two antibodies were selected, AUA1 as specific and HMFG1 as non-specific. Animals were sacrificed 3 days after antibody administration and binding of monoclonal antibodies to tumour and normal tissues was measured. Exposure of the tumours to higher than 40 Gy significantly increased the tumour to blood ratios with both specific and non-specific antibody ($P<0.001$). Specific antibody had approximately a 6-fold *in vivo* binding advantage over the non-specific.

The vascular volume in the tumour and normal tissues was measured using $^{99}Tc^{m}$ $O_4$ red blood cells. Vascular permeability was determined by measuring the amount of $^{125}$I-labelled non-specific antibody extravasated out of the tumour vasculature during 1 h. Vascular volume in the tumour was found to be significantly decreased after exposure to higher doses than 40 Gy ($P<0.001$). Vascular permeability in tumours was higher in all radiation experiments when compared to non-irradiated values but the increases were not significant. We conclude that external irradiation increases specific and non-specific antibody uptake by tumour possibly due to increased permeability, even though vascular volume of the tumour is decreased.

### The detection of gynaecological malignancies by carcinoma associated antigen

S.B. Kelley, L.S. Hanna, N.A. Habib, M.J. Hershman, R.C.N. Williamson & C.B. Wood

*Bristol Royal Infirmary and Departments of Surgery and Obstetrics, Royal Postgraduate Medical School, London, UK.*

Carcinoma associated antigen (CA-50) is a tumour associated carbohydrate antigen, defined by the monoclonal antibody C50, which has been raised against a colorectal adenocarcinoma cell line. The aim of this study was to assess whether CA-50 can differentiate patients with gynaecological malignancies from normal subjects and those with benign disease. A radioimmunoassay was used for the detection of CA-50 in the serum of 50 normal subjects, 26 patients with benign disease and 48 patients with gynaecological malignancies. Serum levels in normal subjects and 21 out of 26 patients (81%) with benign disease were below 17 U ml$^{-1}$, while 27 out of 48 patients (56%) with carcinoma had levels above 17 U ml$^{-1}$. The sensitivities for cervical, endometrial and ovarian carcinomas were 48, 75 and 67% respectively. In patients with cervical carcinoma, the sensitivity was 46% for stage I carcinoma, 36% for stage II and 71% for stages III and IV. Therefore, this test may prove useful in the diagnosis of patients with gynaecological carcinomas.

### Carcinoma associated antigen in the detection of gastrointestinal carcinomas

S.B. Kelly, N.A. Habib & M.J. Hershman

*Department of Surgery, Bristol Royal Infirmary and Department of Surgery, RPMS, Hammersmith Hospital, London, UK.*

Carcinoma associated antigen (CA-50) is a tumour associated carbohydrate antigen, defined by the monoclonal antibody C50, which has been raised against a colorectal adenocarcinoma cell line. The aim of this study was to assess whether CA-50 can differentiate patients with carcinomas, from normal subjects and those with benign disease. A radioimmunoassay was used for the detection CA-50 in the serum of 50 normal subjects, 72 patients with benign disease and 239 patients with carcinoma of the gastrointestinal tract. Serum levels in normal subjects and 67 out of 72 patients (93%) with benign disease were below 17 U ml$^{-1}$, while 157 out of 239 patients (66%) with carcinoma had levels above 17 U ml$^{-1}$. The sensitivities for the various carcinomas were as follows: pancreatic 92% (26 patients), gastric 75% (24), oesophageal 71% (21), liver 66% (91) and colorectal 51% (77). In patients with colorectal carcinoma, the sensitivity was 22% for Dukes' A carcinoma, 29% for Dukes' B, 59% for Dukes' C and 73% for metastatic disease. The CA-50 levels were elevated in 7 out of 9 patients (78%) who developed recurrence following curative surgery. In patients with liver carcinoma, the sensitivity was 70% for cholangiocarcinoma, 54% for hepatocellular carcinoma and 58% for metastatic liver disease. Therefore, this test may prove useful in the diagnosis and monitoring of patients with gastrointestinal carcinomas.

### Carcinoma associated antigen in the detection of urological malignancies

S.B. Kelly, N.A. Habib, M.J. Hershman, R.C.N. Williamson & C.B. Wood

*Departments of Surgery, Bristol Royal Infirmary and Royal Postgraduate Medical School, London, UK.*

Carcinoma associated antigen (CA-50) is a tumour associated carbohydrate antigen, defined by the monoclonal antibody C50, which has been raised against a colorectal adenocarcinoma cell line. The aim of this study was to assess whether CA-50 can differentiate patients with urological carcinomas from normal subjects and those with benign disease. A radioimmunoassay was used for the detection of CA-50 in the serum of 50 normal subjects, 86 patients with benign disease and 104 patients with urological carcinomas. Serum levels in all 50 normal subjects and 83 of 86 patients (97%) with benign disease were below 17 U ml$^{-1}$, while 49 of 104 patients (47%) with carcinoma had levels above 17 U ml$^{-1}$. The sensitivities for prostatic, bladder and renal carcinomas were 43%, 62% and 47% respectively. In patients with prostatic carcinoma, the sensitivities were 0% for well differentiated carcinomas, 33% for moderately differentiated carcinomas, 67% for poorly differentiated carcinomas and 71% for metastatic disease. The sensitivities for non-invasive and invasive bladder carcinomas were 42% and 89% respectively. Therefore this test may prove useful in the diagnosis of patients with urological malignancies.

### Cross-linking of mouse monoclonal antibodies into tetramolecular antibody complexes using rat monoclonal anti-isotype antibodies

P.M. Lansdorp & T.E. Thomas

*Terry Fox Laboratory, BC Cancer Research Centre, Vancouver, Canada.*

Mouse $IgG_1$ monoclonal antibodies can be cross-linked to form cyclic immune complexes using selected rat monoclonal antibodies specific for mouse $IgG_1$. Such complexes are powerful cross-linking reagents as illustrated by their applications for the labelling of monoclonal antibodies with enzymes and fluorescent phycobiliproteins and the specific immunoadsorption of lymphocytes labelled with anti-hapten × anti-lymphocyte tetramers onto columns containing hapten coated glass beads. For these applications tetrameric antibody complexes were prepared with mixtures of two mouse $IgG_1$1 antibodies (e.g. *a* and *b*) and bispecific (e.g. $a \times b$) as well as monospecific ($a \times a$ and $b \times b$) complexes were obtained. The latter compete with bispecific complexes for antigenic sites and thereby reduce the efficiency of the desired bispecific cross-linking. Furthermore, the formation of tetrameric antibody complexes is currently limited to mouse antibodies of the $IgG_1$ subclass. In an attempt to overcome these limitations, rat monoclonal antibodies specific for mouse $IgG_{2a}$ were raised. Several hybridomas secreting antibodies forming highly stable immune complexes were isolated. Thus, tetrameric antibody complexes can now be formed with monoclonal mouse antibodies of both the $IgG_1$ and the $IgG_{2a}$ subclass. The anti-mouse $IgG_{2a}$ hybridomas are now being used in fusion experiments together with rat anti-mouse $IgG_1$ cell lines with the aim of isolating hybrid hybridomas producing bispecific antibodies that specifically cross-link mouse $IgG_1$ to mouse $IgG_{2a}$ in a cyclic tetramolecular complex.

### *In vivo* experimental targeting of lymphocytes with $^{111}$In-labelled monoclonal antibodies

I. Loufti, P.M. Chisholm, A.A. Epenetos, J.R. Batchelor & J.P. Lavender

*Royal Postgraduate Medical School, Hammersmith Hospital, London, UK.*

We have investigated the use of an indium-111-labelled Pan T (MRC OX-19) monoclonal antibody (Mab) in the rat to establish its usefulness in radiolabelling T-cells for imaging and therapy purposes. Biodistribution of the unlabelled Mab after i.v. administration showed early binding to peripheral blood lymphocytes and late detection of cell-bound antibody in lymph nodes and spleen. Modulation of the lymphocyte surface molecule reacting with the antibody in cells obtained from blood, lymph nodes and spleen was observed and was marked when high doses of the antibody were used. This phenomenon was found to be dose dependent. The use of the In-111-labelled Pan T injected i.v. in the rat showed very early splenic localisation which remained high over 24 h, gradual increase in activity in lymph nodes and steady background in muscle, lung and liver. Imaging at 20 h post-injection of the In-111 Pan T revealed uptake in the cervical lymph nodes. This pointed to the usefulness of the technique for *in vivo* use having established the optimal conditions in terms of antibody dose and radioactivity administered.

### The clinical evaluation of tumour marker combinations in the differential diagnosis of benign and malignant liver disease

Michele Lucarotti, N.A. Habib, S.B. Kelly, M.J. Hershman, M.J. Cooper, C.B. Wood & R.C.N. Williamson

*Department of Surgery, Bristol Royal Infirmary and Royal Postgraduate Medical School, London, UK.*

CEA, CA19-9 and CA50 are tumour associated antigens defined by monoclonal antibodies. The aim of this study was to determine whether combined use of CEA, CA19-9 and CA50 could improve diagnostic accuracy.

An immunoradiometric assay was used for the detection of CEA and CA19-9 and the Delfia system for CA50. Serum was collected from 65 normal subjects, 40 with hepatobiliary carcinoma (26 primary, 14 secondary and 17 with benign hepatobiliary disease). The cut-off levels were calculated as the mean of the control group plus 2 standard deviations. The results are shown in the table.

| | *Percentage positive* | | | |
|---|---|---|---|---|
| *Antibody* | *Primary carcinoma* | *Secondary carcinoma* | *Benign* | *Normal* |
| CA19-9 | 46 | 43 | 0 | 9 |
| CA50 | 50 | 64 | 25 | 11 |
| CEA | 20 | 70 | 0 | 8 |

All three antibodies contributed to improving correct classification of secondary liver tumours (multivariant discriminant analysis $P<0.05$) but only CA19-9 and CA50 contributed in primary liver tumour diagnosis (multivariant analysis $P<0.05$). The diagnostic accuracy *vs.* benign disease was 81% for primary and 91% for secondary malignancies. Combined use of CEA, CA19-9 and CA50 may be useful in differentiating benign from malignant hepatobiliary disease.

### The use of tumour marker combinations in the differential diagnosis of patients with pancreatic carcinoma

M. Lucarotti, N.A. Habib, S.B. Kelly, M.J. Hershman, M.J. Cooper, C.B. Wood & R.C.N. Williamson

*Departments of Surgery, Bristol Royal Infirmary and Royal Postgraduate Medical School, London, UK.*

CEA, CA19-9 and CA50 are tumour associated antigens defined by monoclonal antibodies which have been raised against adenocarcinoma cell lines. No single antibody alone is specific for the detection of pancreatic malignancy and the aim of this study was to determine whether the combined use of CEA, CA19-9 and CA50 would improve diagnostic accuracy. An immunoradiometric assay was used for the detection of CEA and CA19-9 and the Delfia system for DA50. Serum was collected from 65 normal subjects, 16 with pancreatitis and 28 with pancreatic carcinoma and stored at −20°C until analysis. The cut-off levels (COL) were calculated as the mean of the control group plus two standard deviations. The results are shown in the table.

| | *Percentage positive* | | |
|---|---|---|---|
| *Antibody* | *Carcinoma* | *Pancreatitis* | *Normal* |
| CA19-9 | 85 | 20 | 9 |
| CA50 | 92 | 19 | 11 |
| CEA | 37 | 13 | 8 |

In order to improve the specificity we used multivariant discriminant analysis on the combination of antibodies. This showed that 96% of the malignant group, 13% of the pancreatitis group and 11% of the normal group were positive with an overall correct classification of 91% into the three groups (multivariant discriminant analysis $P<0.05$). Combined use of CEA, CA19-9 and CA50 improves diagnostic accuracy and may be useful in differentiating benign from malignant disease.

### Immunohistochemical characterisation of a breast cancer associated antigen using BCD-F2 monoclonal antibody

Rosemonde Mandeville, Salwa Sidrac-Ghali & Lise Giroux

*Immunological Research Center, Institut Armand-Frappier, Laval-des-Rapides, Quebec, Canada H7N 4Z3.*

Monoclonal antibodies (MAbs) were produced in mice immunised by BT-20, a human breast adenocarcinoma cell line. The screening of these MAbs was performed by both ELISA and indirect immunofluorescence (IF) techniques using breast cancer cell lines and fresh frozen tissues. Hybridomas recognising BT-20 cells but negative with HBL-100 (putative normal epithelial cell line of breast origin) were retained for further studies. The biochemical characterisation of one of these MAbs, BCD-F2, has been determined and showed that it recognises a breast cancer associated antigen of $M_r$ 48,000 which appears to be a *N*-acetyl-D-glycosamine carbohydrate side chain on a glycoprotein. In the present study using immunohistochemical techniques, BCD-F2 was shown to react with 98% (92% strongly and 6% weakly) of primary breast carcinomas. BCD-F2 also reacted with 82% (32% strongly and 50% weakly) of carcinomas from extra-mammary origin and did not recognise sarcomas, lymphomas or seminomas. These results suggest that BCD-F2 has potential clinical use in (a) the differential diagnosis between carcinomas from sarcomas and lymphomas, (b) the detection of micro-metastases in lymph nodes and (c) the identification of neoplastic cells in fine needle aspirates and biopsies.

### Predicting targeting results in ovarian cancer

L. Massuger, R. Verheijen, P. Peelen, L. Poels, O. Boerman, C. Yedema & P. Kenemans

*Departments of Obs. and Gyn., Histology and Urology, St Radboud Hospital, University of Nijmegen, Free University, Amsterdam, The Netherlands.*

Both accessibility of the tumour associated antigens (TAAs) and binding of radiolabelled monoclonal (MAbs) to these TAAs determine the outcome of diagnostic and therapeutic immunotargeting in ovarian cancer. In order to gain information on the amount of antibody–antigen binding we developed a new method to quantitate the *in vitro* MAb binding in tissue sections of 12 epithelial ovarian tumours. An automated image analysis system, Omnicon FAS II, was used to quantitate the area of a tumour section stained with different MAbs using standard immunoperoxidase techniques. In order to assess the tumour associated marker *vs.* epithelial marker ratio, tissue sections were stained with monoclonal OV-TL 12-5 (intermediate filament antibody specific for epithelial tissue) and the ovarian cancer associated MAbs OC125 and OV-TL 3.

In the tumour samples studied, OV-TL 3 tumour cell binding percentages (OV-TL 3/OV-TL 12-5) ranged from 46 to 137% with a mean of 79.6% whereas OC125 ranged from 4 to 85% with a mean of 50.5%. Therefore OV-TL 3 seems to be a better candidate for *in vivo* diagnostic and therapeutic targeting in the majority of patients in our study.

This new method of quantitation of antibody–antigen binding seems to be accurate and reproducible and could be used to predict clinical targeting results.

### Immunoscintigraphy in patients with melanoma

C.L. Mulder, P. Hol, C.A. Hoefnagel, J. Hilkens & P.F. Bruning

*Division of Tumour Biology, The Netherlands Cancer Institute (Antoni van Leeuwenhoekhuis), Amsterdam, The Netherlands.*

We have performed immunoscintigraphy in patients with melanoma using $^{99}Tc^{m}$-labelled $F(ab')_2$ fragments of MAb 225.28S and $^{131}I$-labelled MAb M6 (IgG1). Both MAbs are directed against a high molecular weight melanoma associated antigen.

We have injected MAb 225.28S in 18 patients with metastatic melanoma. In 8 out of the 18 patients there was uptake of the labelled MAb by tumour lesions. In organs with a high non-specific uptake, like liver and spleen, tumour localisations were seen as cold spots. $^{131}I$-labelled M6 was administered to 4 patients with metastatic melanoma. In one patient most of the cutaneous and muscular metastases showed uptake, but pulmonary and abdominal tumour localisations did not. In another patient there was uptake in the liver metastases in contrast to a mediastinal mass.

In two other patients no uptake of labelled MAb was observed in any of the tumour lesions. In one patient there was a false positive concentration of labelled MAb in the left femur.

We conclude that immunoscintigraphy with MAb 225.28S has a low sensitivity and that MAb M6 may prove superior for imaging.

### *In vivo* scanning with radiolabelled UJ13A in medullary carcinoma of thyroid

A. O'Meara, R.J. Fitzgerald, P. Dervan, J. Kingston, L. Latchford & J. Kemshead

*Children's Research Centre, Our Lady's Hospital for Sick Children, Dublin, Ireland, Department of Pathology, Mater Misericordiae Hospital, Dublin, Ireland and ICRF, London, UK.*

An 8-year-old girl presented with medullary carcinoma of thyroid metastatic to cervical nodes on right side of neck. Following total thyroidectomy and block dissection of right cervical lymph nodes (Nov. 1986) serum calcitonin remained elevated at 2,000–2,500 $ng\,l^{-1}$ (preop. 12,000 $ng\,l^{-1}$).

To detect evidence of occult disease, postoperative investigations included pentavalent DMSA (−ve) and MIBG (−ve). Being a tumour of neuroectodermal origin, tumour tissue was screened with a panel of monoclonal antibodies to cells of neuroectodermal origin and demonstrated strong reactivity to UJ13A. In view of this, she was given an *in vivo* dose 3 mCi $^{123}I$ UJ13A and this demonstrated slightly increased uptake in the left side of the neck. Three months after radioscintigraphy serum calcitonin was 300 $ng\,l^{-1}$. There is no clinical evidence of disease.

Given the nature of the isotope and the low dose of radiation administered it is conceivable that the reduction in serum calcitonin could have been mediated via an ADCC reaction.

### Immunoscintigraphy (IS) with $^{131}I$-labelled HMFG2 and HMFG1 $(Fab')_2$ *vs.* abdominal CT scan in the detection of residual disease in ovarian cancer

D. Pectasides, K. Pateniotis, L. Tzimis, X. Trapali, P. Natsis, P. Arapantoni, J. Taylor-Papadimitriou, A. Epenetos, P. Koutsiouba & A. Athanassiou

*Anticancer Hospital, Greece and ICRF, London, UK.*

$^{131}I$-labelled MoAb HMFG2 and HMFG1 $F(ab')_2$ were administered intraperitoneally in 15 patients with ovarian cancer who had completed their chemotherapy. Each patient received 2–3 mCi. Patients were scanned at 24–96 h post-injection. In 3/15 patients IS failed because adhesions prevented diffusion of the MoAb. Of the remaining 12 patients 9 underwent second look laparotomy (SL). Results are shown in the following table.

| | *Positive* | *Negative* |
|---|---|---|
| SL | 8 | 1 |
| IS | 9 | 0 |
| CT | 6 | 3 |

IS was true positive in 8/9 (90%) patients whereas CT in 6/9 (67%) patients. Of the 3 patients not undergoing SL, IS was positive in all of them whereas clinical examination and abdominal CT scan were negative. All 3 patients had subsequent clinical relapse at 3, 4 and 5 months. The IS true positivity was 92% (11/12 patients) whereas CT was 50% (6/12%).

We conclude that IS with intraperitoneal administration of $^{131}I$-labelled MoAb can accurately detect the presence of residual disease in ovarian cancer. In this pilot study it proved more sensitive than abdominal CT scan.

**Localisation of biotinylated monoclonal antibodies in nude mice bearing subcutaneous and intraperitoneal human tumour xenografts**

S. Pervez, G. Paganelli, A.A. Epenetos, W.J. Mooi, D.J. Evans & T. Krausz

*Department of Histopathology and ICRF Oncology Laboratory, Hammersmith Hospital, London, UK.*

To assess the precise distribution of *in vivo* injected mouse monoclonal antibodies in nude mice on histological sections, one can use radiolabelled antibodies for autoradiography, or employ immunohistochemical methods. Autoradiography, though very useful, is time consuming and does not provide a precise picture of the cellular localisation of the antibody. With conventional immunohistochemical techniques there is also possible interaction of the anti-mouse antibody with the host tissue antigens.

To overcome this problem we have biotinylated our primary antibodies (AUA1 and CEA) and injected each separately intra-peritoneally into nude mice bearing simultaneous subcutaneous and intraperitoneal xenografts of the human tumour 'LOVO' (a cell line established from an adenocarcinoma of the colon). Twenty-four hours after injection, the animals were sacrificed, tumours and control organs were studied. Antibody was demonstrated on frozen sections by incubating sections with avidin biotin peroxidase complex.

We compared the *in vivo* penetration and distribution of these antibodies on intraperitoneal and subcutaneous tumours as well as control organs. The antibody penetration was restricted to the thin layer of tumour cells adjacent to the vascular stroma in large solid subcutaneous and intraperitoneal tumours, whereas in very small intraperitoneal tumours, antibody penetration was complete. These findings were similar to our autoradiographic results.

This study demonstrates that employing biotinylated antibodies for *in vivo* localisation studies provides superior resolution of antibody binding for morphological assessment compared to autoradiography. Localisation of a biotin label is more precise and will permit ultrastructural studies.

**Evaluation of serum CA 125 levels in the monitoring of response to chemotherapy in epithelial ovarian carcinoma (EOC)**

M. Quaranta, M. Coviello, G. Micelli, A. Casamassima, I. Abbate, M. Correale & V. Lorusso

*Institute of Oncology, Bari, Italy.*

Recently, a monoclonal antibody-based immunoassay (EIA) for CA 125 antigen has been used to monitor the treatment of EOC. Serum CA 125 levels were evaluated in 60 patients, mean age 56 (30–69 years), with ovarian cancer. CA 125 reference limit was $35\,U\,m^{-1}$, as proposed by Bast. Serial CA 125 measurements were assessable in 23 patients undergoing chemotherapy. Rising, falling and unchanged levels correlated with disease in 21 of 23 cases (95%). Ten out of eleven patients who showed objective response to chemotherapy had a decrease in antigen levels. Two out of three patients with stable disease had unchanged levels, and progression ($n=9$) was always associated with rising levels. Mean value of decreased levels was 65% (range 30–95%).

| | *Antigen levels* | | |
|---|---|---|---|
| *Disease status* | *Decreased* | *Unchanged* | *Increased* |
| Complete response | 3 | – | – |
| Partial response | 7 | 1 | – |
| Stable disease | 1 | 2 | – |
| Progression | – | – | 9 |

These data suggest that CA 125 may aid in early identification of non-responders. However, a normal CA 125 level does not exclude the presence of disease.

**Intravenous EDTA to reduce bone uptake of $^{90}Y$ following $^{90}Y$-labelled antibody administration**

Gail Rowlinson, D. Snook, S. Stewart & A.A. Epenetos

*ICRF Oncology Group, Hammersmith Hospital, London, UK.*

Marrow toxicity due to bone uptake of $^{90}Y$ is the dose-limiting factor in radioimmunotherapy using $^{90}Y$-labelled monoclonal antibodies. Using a human tumour xenograft model, we have investigated the efficacy of EDTA (ethytenediaminetetraacetic acid) in removing free $^{90}Y$ from the circulation, but not from the antibody.

Nude mice with intraperitoneal tumours of the human colon carcinoma, LOVO, were injected intraperitoneally with $^{90}Y$-labelled AUA1 antibody. Half of the animals received 20 mg EDTA intravenously 2 h after antibody administration, and then every 24 h until dissection. Percentage of injected dose $g^{-1}$ and tumour to normal tissue ratios were compared in the two groups of mice.

Two days after antibody administration the tumour/bone ratios (mean ± s.d.) in untreated mice was $3.6 \pm 1.1$, compared with $7.8 \pm 1.6$ in EDTA-treated mice ($P<0.05$). Of the other tumour/normal tissue ratios, only kidney showed any significant difference (control $2.0 \pm 0.5$ *vs.* treated $4.0 \pm 1.0$, $P<0.05$). The percentage of injected dose $g^{-1}$ of tissue in blood, tumour and all normal organs except bone and kidney was not significantly different, indicating that the $^{90}Y$ was not removed from the bound and circulating antibody.

Excretion of $^{90}Y$ in the urine of EDTA-treated mice was 2.7 times higher than in controls at 18 and 24 h after antibody injection. There was no significant difference at 48 h by which time most of the circulating free $^{90}Y$ had been chelated by the EDTA and excreted.

These studies indicate that EDTA may be used to reduce $^{90}Y$ toxicity, without adversely affecting the antibody-guided radiation dose to tumour.

**Combination of immunoscintigraphy and CA125 serum levels in ovarian cancer**

Ch. Schatten, N. Pateiskey, P. Sevelda, K. Czerwenka, M. Barrada, K. Philipp & J. Burchell

*First Department of Obstetrics and Gynaecology, Vienna, Austria and ICRF, London, UK.*

Forty-three patients with a history of ovarian cancer were investigated prior to second-look surgery using immunoscintigraphy (IS) and determinations of CA125 serum levels. While IS provide information concerning the localisation of tumour sites, CA125 serum levels $\geqslant 35$ IU served as an indicator for recurrent disease. For IS an IgG1 mouse monoclonal antibody HMFG2, raised against human-milk-fat-globule and expressed on the cell surface membrane of ovarian cancer cells, was employed after labelling with $^{123}I$. In 27 out of 30 cases IS proved to be true positive and in 9 out of 13 cases true negative. In 29 patients CA125 serum levels were also assessed. The combination of both methods showed superior results than each method alone. In this case the value of sensitivity could be raised to 0.94, specificity to 1.00, accuracy 0.96, positive prediction 1.00 and negative prediction 0.88, respectively.

### Induction of tolerance to xenogeneic immunoglobulins

G.B. Sivolapenko, R.J. Edwards, M. Kanariou, N.S. Courtenay-Luck, A.A. Epenetos & M.A. Ritter

*Hammersmith Hospital, London, UK and Aghia Sophia Hospital, Athens, Greece.*

Humoral responses against passively administered immunoglobulins had always been a problem for all kinds of serotherapy. We attempted to reduce this response by carefully deaggregating (Superose 6 or 12, on FPLC) immunoglobulins.

When deaggregated, polyclonal human IgG was injected i.p. into Balb/c mice, there was no significant reduction in the primary anti-human IgG response, whereas with CBA and C57BL/6 there was a reduced response. A second injection boosted the humoral response of the Balb/c, whereas CBA and C57BL/6 responded only partially. When Cyclosporin A was administered to Balb/c, we obtained a reduction in responses. When rats (Lou/c) and rabbits (1/2 lop) were immunised with a deaggregated mouse monoclonal antibody, we did not obtain any difference in their anti-mouse IgG response compared to controls. In a group of 10 patients with cancer we administered deaggregated $^{131}$I-labelled mouse monoclonal antibody and in another group of 10 patients $^{90}$Y-labelled antibody, which was aggregated during the radiolabelling procedure. We obtained no difference in the humoral responses against mouse IgG between those 2 groups.

Our data, therefore, show that deaggregation of xenogeneic IgG does not prevent the host's humoral anti-Ig responses.

### Biodistribution of $^{111}$In OC 125 monoclonal antibody (MABG) in nude mice bearing intraperitoneal human ovarian carcinoma

Ph. Thedrez, M. Kremer, C. Curtet, C. Sai, D. Guerreau, J.C. Saccavini & J.F. Chatal

*INSERM U 211, Nantes and Comp. ORIS Ind., Grif sure Yvette, France.*

Intact MAB OC 125 with high specificity for ovarian carcinoma was labelled with $^{111}$In or $^{131}$I and injected intraperitoneally or intravenously into nude mice bearing human ovarian carcinoma NIH:OVCAR-3 in order to compare biodistribution according to the radiolabel used and route of administration. Mice were sacrificed at: 2 h, 24 h, 96 h and 7 days. Results are expressed in % of injected dose per gram and in tumour-to-tumour ratios.

Tumour uptake 24 h after i.p. injection was higher with $^{111}$In OC 125 ($28 \pm 7.44$) than with a non-specific immunoglobulin ($^{111}$In NS) ($6.86 \pm 1.35$). The kinetics of uptake also differed, showing a plateau followed by a drop at day 7 with $^{111}$In OC 125 and a decrease beginning at 24 h with $^{111}$In NS. Tumour-to-normal-tissue ratios ranged between $29 \pm 1.10$ and $4 \pm 0.84$ with $^{111}$In OC 125 and between $9.48 \pm 6.82$ and $0.89 \pm 0.29$ with $^{111}$In NS. After intravenous injection, the maximum uptake in tumour with $^{111}$In OC 125 was only $14.6 \pm 2.7$ at 96 h, with a lower tumour-to-normal tissue ratio. The i.p. injection of $^{131}$I-OC 125 showed lower uptake in tumour ($13.5 \pm 4.2$, $4.8 \pm 3.2$, $3.5 \pm 1.3$ and $0.3 \pm 0.1$ at the different time intervals) but with tumour-to-tissue ratios equal to those obtained with $^{111}$In OC 125. Biodistribution of i.p.-injected radiolabelled colloids was also performed and showed maximum tumour uptake at 96 h ($20.2 + 5.3$) but with elevated non-specific uptake in liver ($31.0 \pm 6.2$) and spleen ($55.2 \pm 14.1$). These results indicate high and selective tumour uptake of $^{111}$In OC 125 after i.p. injection and suggest an i.p. radioimmunotherapy could be carried out by replacing $^{111}$In with $^{90}$Y.

### Imaging thrombus with radiolabelled Fab′ fragments of a monoclonal antibody to platelets

A.W.J. Stuttle, C.J. O'Donnell, N. Virji, A.M. Peters, R.C. Harrison, G.J.P. Murphy & J.P. Lavender

*Hammersmith Hospital, London and Amersham International, UK.*

A simple rapid method, not requiring blood cell separation, is required for platelet labelling in the diagnosis of DVT. We have previously reported our preliminary clinical findings with an i.lv. monoclonal antibody (P256), raised in mice labelled with $^{111}$In and specific for primate platelet glycoprotein IIb/IIIa complex. The effect of P256 and its Fab; fragments on *in vitro* platelet function have been assessed by measuring platelet aggregation in whole blood, platelet rich plasma and gel filtered platelets. The $F(ab')_2$ fragment caused consistently less aggregation than the whole antibody, whilst the monovalent Fab′ fragment had no effect. The monovalent Fab′ fragment was therefore investigated for its ability to image thrombus *in vivo*. After labelling with $^{111}$In, via the bifunctional chelate DTPA, the P256 Fab retained reactivity and specificity for primate platelets. Thrombus was induced in *M.fascicularis* by i.v. injection of iron microspheres and localised with an externally applied magnetic field. Thrombus was confirmed by venography and imaged with $^{111}$In-P256 Fab′. The venogram and scintigram were both positive at 1 and 48 h after thrombus induction. These initial studies show good correlation between imaging with P256 Fab′ fragment and venography for detection of DVT.

### Monoclonal antibodies against mesenchymal antigens of the human mammary gland

A.A. Verstraeten, Ph.C. Hageman & J.H. Daams

*Division of Tumourbiology, Antoni van Leeuwenhoekhuis, Plesmanlaan 121, Amsterdam, The Netherlands.*

Interactions between epithelial and stromal components are known to be involved in epithelial carcinogenesis of the mammary gland. Tenascin, an extracellular matrix (ecm) protein with a hexabrachion (Mackie *et al.*, P.N.A.S. **84,** 4621, 1987.

In search of markers for ecm-components of human mammary tumours monoclonal antibodies (moabs) were raised against a stroma-enriched preparation of a human mammary carcinoma specimen and selected, by immunohistochemistry on frozen sections of normal mammary glands and mammary tumours, for their reactivity with tumour stroma. Two moabs, T3C8 and T2H5, fulfilled the criteria and were further characterised. The reaction pattern of T3C8 closely resembles the pattern obtained with rabbit-anti-tenascin-antiserum (kindly provided by Dr. E.J. Mackie), i.e. a clearly positive staining of tumour-stroma, especially on the border tumour/stroma, and also around normal pre-existing ducts and alveoli and in the wall of blood vessels. The reaction pattern of T2H5 was very similar to T3C8; however, no reaction was seen around normal ducts and alveoli. The stroma of fibroadenomas was positive with both antibodies.

As yet no biochemical data are available on the biochemical nature of the molecules recognised by these two moabs, nor whether they are related to tenascin or other ecm-components. The distribution pattern of these moabs, however, demonstrates its usefulness in studying the role of ecm in tumour formation.

### Immunoscintigraphy of colorectal cancer using indium labelled monoclonal antibody 77.1

C.Y. Yiu, Louise Baker, B.R. Davidson, K. Roberts, Gill Clarke, R. McCready, J. Westwood, P.B. Boulos & C.G. Clark

*University College, London, Royal Marsden Hospital and Institute of Cancer Research, London, UK.*

77.1 (IgG2a), a monoclonal antibody raised against bladder cancer was found to react with colorectal cancer on immunocytochemistry. Xenograft localisation studies confirmed localisation of 77.1 to target tumour. An immunoscintigraphy study was performed using $^{111}$In-labelled 77.1. Fourteen patients with 15 tumours took part in the study. Two of patients had recurrent disease. $^{111}$In-labelled antibody was injected intravenously and scanning was performed at 3–5 days after injection. Immunocytochemistry was performed on resected tumours. Positive scans were obtained for 10 tumours (64%) in 10 patients. Surgical specimen was not available for staining for one patient who had a positive scan. All patients with positive scans had positive immunocytochemistry. The tumours from three patients with negative scans stained positively with 77.1. One patient whose tumour stained negatively with 77.1 also had a negative scan. Analysis of specimens resected six days following antibody administration revealed a mean tumour to normal colon ratio of 1.93 (range 1.0–3.26) and a mean tumour to blood ratio of 3.55 (range 1.6–5.07). These results are similar to those obtained with other antibodies used in immunoscintigraphy of colorectal cancer and suggest that further evaluation of this antibody is indicated.

### Immunoscintigraphy of colorectal cancer with an antibody reactive with epithelial membrane antigen (EMA)

C.Y. Yiu, L. Baker, B.R. Davidson, M. Ward, K. Roberts, G. Clarke, C. Ward, J. Westwood, P.B. Boulos & C.G. Clark

*Departments of Surgery and Nuclear Medicine, University College, Royal Marsden Hospital, St George's Hospital and Institute of Cancer Research, London, UK.*

Immunoperoxidase staining of LICR-LON M8, a mouse monoclonal antibody reactive with epithelial membrane antigen (EMA) showed reaction with sections of colorectal cancer. $^{111}$In-labelled M8 has already been used in the immunoscintigraphy of breast cancer. These findings prompted an immunoscintigraphic study of colorectal cancer patients with $^{111}$In-labelled M8. Eighteen patients took part in the study. Histological sections were available from 16 patients for immunocytochemistry. $^{111}$In-labelled M8 was injected intravenously and scanning took place at 3–5 days. Positive scans were obtained from 13 patients (65%). All tumours with positive scans had positive staining with M8. The tumours from 2 patients with negative scans also had negative staining with M8. Three patients had positive immunocytochemistry but negative scans. The surgical specimen available from one patient at day 4 showed a tumour blood ratio of 3.5. Eleven patients had samples taken of tumour and normal tissues on day 6. The ratio of antibody uptake of tumour to normal colon was $2.56 \pm 1.27$ (mean $\pm$ s.d.) and the ratio of tumour to blood activity was $11.0 \pm 6.2$ (mean $\pm$ s.d.). These results suggest that there is specific uptake of $^{111}$In-labelled M8 by tumours.

### Immunohistochemistry with monoclonal antibodies to a sialomucin in adenolymphomas of the parotid gland: a novel method for epitope recognition

St. Zotter, Ph. Hageman, A. Lossnitzer, Jvd. Tweel, J. Hilkens, W. Mooi & J. Hilgers

*Institute of Pathol. Anatomy, Dresden, DDR, Departments of Tumour Biology, Pathology, NKI and Department of Obs. & Gyn., VU, Amsterdam, The Netherlands.*

Fourteen monoclonal antibodies (MoAbs) to the epithelial membrane antigen (EMA) complex on a sialomucin molecule were studied for their immunohistochemical reactivity with serial sections of fourteen formalin-fixed and paraffin-embedded adenolymphomas of the parotid gland. Two types of reactivity were observed, suggesting different cellular deposition of the corresponding epitopes. Most antibodies reacted with the luminal membrane of an upper layer of epithelial tumour cells (type A reaction). The other reaction (type B) was observed with the membrane of basal epithelial cells. The antibodies could be ranked according to their tendency to show type A and/or type B reactions. MoAb Cal was the only one with a pure type A reaction. A strong preference of type A reactivity (with traces of type B reactions) was observed for the antibodies HMFG-2, M8, E29 and NCRC-11. Several antibodies gave good type B reactions in addition to strong A type reactivity (MoAbs 126E7, 115G2, 115D8, 140C1, F36/22, 139H2). MoAb DF3 showed equally strong reactions with both cell types. A clear-cut preference of the reactions with basal cells was seen with the antibodies HMFG-1 and 115F5. The subclassification of the antibodies does fit epitope mapping, by conventional blocking studies, reported in the literature.